# Treatment Outcomes of Children with Primary Versus Secondary Callous-Unemotional Traits

**DOI:** 10.1007/s10802-023-01112-6

**Published:** 2023-08-08

**Authors:** Georgette E. Fleming, Bryan Neo, Silvana Kaouar, Eva R. Kimonis

**Affiliations:** Parent-Child Research Clinic, School of Psychology, The University of New South Wales, Sydney, New South Wales Australia

**Keywords:** Callous-unemotional traits, Variants, Parent training, Psychopathic traits

## Abstract

Objective: Recent efforts to improve outcomes for young children with conduct problems and callous-unemotional (CU) traits involve adapting treatments to meet the unique needs of this subgroup. However, these efforts have ignored accumulating evidence for distinct primary and secondary variants within the CU subgroup. Existing treatment adaptations uniformly target risk factors associated with primary CU traits and no studies have investigated variant-specific patterns of responsiveness to treatment adaptations among young children with CU-type conduct problems. Method: Participants were 45 families with a 3- to 7-year-old clinic-referred child (*M* = 4.84 years, *SD* = 1.08, 84% boys) with conduct problems and CU traits. Primary and secondary CU variants were defined based on baseline parent-rated anxiety scores. All families received Parent-Child Interaction Therapy adapted for CU traits (PCIT-CU) at an urban university-based research clinic. Families completed five assessments measuring child conduct problems and affective outcomes. Results: Linear mixed-effects modeling showed that the rate and shape of change over time in conduct problems differed between variants, such that children with secondary CU traits showed deterioration in defiant and dysregulated behaviors from post-treatment to follow-up, whereas primary CU traits were associated with maintained gains. There were no variant differences in rate of improvement in CU traits. Affective empathy did not improve for either variant. Internalizing problems meaningfully improved by follow-up for children with secondary CU traits. Conclusions: Findings suggest that PCIT-CU is a promising intervention for children with conduct problems and primary CU traits, but may require further personalization for children with secondary CU traits. This trial was registered with the Australian New Zealand Clinical Trials Registry (ACTRN12616000280404).

**Supplementary Information:**

The online version contains supplementary material available at 10.1007/s10802-023-01112-6.

Children with conduct problems are a heterogeneous group. Researchers have attempted to parse this population into more homogenous subgroups via subtyping, whereby certain characteristics identify children with distinct etiological profiles who may differ in prognostic risk. Subtyping according to the presence of callous-unemotional (CU) traits has proven useful for designating a subgroup of children with early-onset, stable, and severe conduct problems who are at considerable risk for poor outcomes in later life (Frick et al., 2014; Neo & Kimonis, 2021). This subtyping approach was codified in the fifth edition of the Diagnostic and Statistical Manual of Mental Disorders (DSM-5; American Psychiatric Association, 2013) as a specifier for Conduct Disorder (CD) called ‘with limited prosocial emotions’ (LPE), given when a child meets criteria for CD and also shows two or more of the following symptoms persistently across multiple settings or relationships: [a] lack of remorse or guilt, [b] callous-lack of empathy, [c] lack of concern about performance, and [d] shallow/deficient affect. A key contribution of this diagnostic specifier is its role in treatment-planning: Research supports that young children with CU traits may be less likely to show normalization of conduct problems following traditional interventions (Perlstein et al., 2023). Instead, they require intensive treatment tailored to their unique emotional, cognitive, and familial characteristics (Frick, 2012). However, the CU intervention literature wholly neglects compelling evidence for distinct variants within the CU subgroup. Extrapolating from the adult psychopathy literature, various researchers have proposed that CU traits can be further divided into primary or secondary variants, with secondary CU traits characterized by comorbid anxiety or history of trauma exposure (Craig et al., 2021). Despite accumulating research on the efficacy of treatments for young children with conduct problems and CU traits, no studies have explored variant-specific patterns of treatment responsiveness. We aim to address this gap by testing whether patterns of responsiveness to a targeted intervention differ among young children with conduct problems and primary versus secondary CU traits.

CU traits are the putative developmental precursor to the affective dimension of psychopathy, which is a multidimensional personality disorder characterized by a narcissistic and manipulative interpersonal style; shallow and deficient affect; and impulsive and antisocial behavior (De Brito et al., 2021). Psychopathy variants were first introduced by Karpman (1941), who proposed that etiology distinguished “symptomatic” (secondary) psychopathy from “idiopathic” (primary) psychopathy. Phenotypically indistinguishable, the latter lacked the ‘psychogenesis’ that characterized the former. Specifically, secondary psychopathy represented a ‘character neurosis’ born from an environmental cause, typically a disruption in the parent-child relationship, which undermined conscience development (Karpman, 1948). In contrast, primary psychopathy was ‘constitutional,’ marked by lack of conscience and corresponding neuroses (Karpman, 1948). Porter (1996) offered an alternative etiological account of secondary psychopathy, proposing that traumatic interpersonal experiences during childhood can ‘de-activate’ the emotional system to promote coping via ‘emotional numbing’. With repeated exposure to trauma or maltreatment, this pattern of dissociation disturbs the developing conscience, resulting in psychopathy acquired through learning (Porter, 1996). Modern perspectives of childhood CU traits typically reflect elements of one or both accounts, whereby secondary CU variants are identified based on elevated CU traits that co-occur with high anxiety or, less frequently, trauma history (Craig et al., 2021).

A burgeoning empirical literature supports the validity of childhood CU variants. A recent systematic review reported that most studies found evidence for clinically meaningful and theoretically consistent CU or psychopathy variants across community, clinical, and justice-involved samples of children and adolescents from ethnically diverse backgrounds (Craig et al., 2021). Despite some inconsistencies, the weight of cross-sectional evidence supports that children with secondary CU traits show a more complex and severe clinical profile than the primary variant (Craig et al., 2021). Critically, a small but accumulating number of longitudinal studies have considered variant-specific differences in outcomes over time, almost uniformly demonstrating that secondary CU traits are associated with worse prognosis across multiple domains relative to the primary variant. Findings are most consistent for internalizing psychopathology, with longitudinal studies reliably demonstrating that secondary variant groups established at various ages during childhood show significantly greater internalizing problems in later life using both normative (Ezpeleta et al., 2017; Fanti & Kimonis, 2017; Goulter et al., 2021) and high-risk clinical (Bégin et al., 2021) samples. There is also some, albeit less consistent, evidence that secondary CU traits are associated with more severe externalizing problems across the lifespan relative to primary CU variants (Ezpeleta et al., 2017; Goulter et al., 2021), as well as poorer relational, cognitive, academic, and vocational outcomes (Fanti & Kimonis, 2017; Goulter et al., 2021).

Taken together, these longitudinal studies provide preliminary support for the predictive—and thus clinical—utility of parsing CU variants, showing that co-occurring CU traits and anxiety assessed as early as three years are associated with moderate stability and considerable risk for numerous, varied, and severe outcomes into adulthood. However, there is also robust evidence that CU traits are not immutable, but influenced by both genetic and environmental factors, including parenting practices (Hyde et al., 2016). Accordingly, there is a clear role for effective, early intervention for children with secondary CU traits. To date, however, no studies have tested whether young children with this specific constellation of behavioral and affective characteristics respond to targeted psychosocial interventions.

The broader literature indicates that children with CU traits benefit from intervention, but begin and end treatment with more severe conduct problems that may continue to meet or exceed diagnostic thresholds, compared to children with conduct problems alone (Fleming, 2023; Perlstein et al., 2023). This literature recognizes that etiology differentiates children with conduct problems with and without CU traits, proposing that traditional interventions do not adequately address the unique emotional, cognitive, and familial risk factors involved in the development and maintenance of conduct problems when CU traits are elevated (Frick, 2012). Accordingly, treatment adaptations for children with CU traits specifically target one or more of these key risk factors (Fleming, 2023). For example, each published treatment adaptation incorporates strategies for improving children’s emotional skills, based on research showing that children with CU traits show unique deficits in their neurological, psychophysiological, and cognitive-behavioral responses to socioaffective stimuli (Frick et al., 2014), which are identifiable from early infancy (e.g., Bedford et al., 2015; Wagner et al., 2020). This multilevel socioemotional insensitivity is targeted in treatment adaptations by augmenting traditional behavioral modification approaches with modules that train children in various emotional skills (e.g., emotion recognition; Dadds et al., 2012, 2019; Fleming and Kimonis, 2018; see also White et al., 2022). Other risk factors implicated in the etiology of CU traits, and thus targeted in treatment adaptations, include low parental warmth and a reward-oriented, punishment-insensitive learning style related to temperamental fearlessness (Fleming & Kimonis, 2018; Waschbusch et al., 2020).

However, these treatment adaptations were specifically designed to target risk factors associated with primary CU traits. Critically, it is unclear whether they also address the risk factors involved in the development and maintenance of secondary CU traits, giving rise to the possibility that the adaptations may not be equally effective for all members of the CU subgroup. Indeed, many modern theories of secondary psychopathy/CU traits position temperamentally-based emotional *hyper*sensitivity as critical to the development of secondary CU traits, whereby childhood trauma or maltreatment interacts with the child’s disposition to lead to dissociation or overarousal, causing children to miss others’ emotional cues and thus fail to develop emotional skills over time (Kimonis, 2023). This developmental model highlights two key risk factors for secondary CU traits: Exposure to trauma or maltreatment and—in contrast to developmental models of primary CU traits—impaired emotional processing characterized by hypersensitivity and dysregulation in response to socioaffective stimuli (Craig et al., 2021).

Regarding trauma or maltreatment exposure, none of the adapted programs explicitly address trauma responses or associated anxiety symptoms. However, some adaptations are to interventions classified as trauma-informed, with accumulating evidence for their efficacy in reducing conduct problems, trauma symptoms, and future maltreatment incidents among children exposed to abuse and neglect (e.g., Parent-Child Interaction Therapy [PCIT]; Gurwitch and Warner-Metzger, 2022). Moreover, most existing treatment adaptations are nested within parent training programs such as PCIT, which emphasize the importance of reducing harsh, inconsistent parenting practices to disrupt coercive cycles of parent-child interaction. While standard training programs employ strategies based on the principle of differential attention (e.g., strategic ignoring of attention-maintained behaviors, specific praise for prosocial behaviors), some CU adaptations also explicitly emphasize the importance of increasing parental warmth (e.g., Fleming and Kimonis, 2018). Since children with both primary and secondary CU traits experience more risky parenting than typical children (Ezpeleta et al., 2017; Fanti & Kimonis, 2017; Meehan et al., 2017) and possibly those with conduct problems alone (Bégin et al., 2021; Fanti & Kimonis, 2017), adaptations targeting parenting practices will likely benefit both CU variants.

Regarding emotional hypersensitivity and dysregulation, it is possible that the central focus of treatment adaptations on improving empathy by enhancing sensitivity to others’ distress cues (Dadds et al., 2012, 2019; Fleming et al., 2022; Kimonis et al., 2019; White et al., 2022) may fail to address or even exacerbate emotional *hyper*sensitivity as a key maintaining factor of secondary CU traits. On the other hand, standard parent training programs can improve child emotion regulation outcomes (e.g., Rothenberg et al., 2019), likely via treatment-related improvement in children’s conduct problems following parental use of reinforcement-based strategies (i.e., ignoring, praise), as well as parental use of positive emotion socialization strategies (Rothenberg et al., 2019). Since treatment adaptations for CU traits retain these elements of their standard progenitors, it is possible that the dysregulation characteristic of secondary CU traits is similarly responsive to treatment.

Taken as a whole, this literature offers a murky picture regarding possible variant differences in treatment responsiveness. It sheds little light on whether we can expect treatment adaptations specifically developed to address the risk factors involved in the development of primary CU traits to be more, less, or equally effective for young children with secondary CU traits. In lieu of empirical guidance, predictions based on theory warrant consideration. Both Karpman and Porter argued that secondary psychopathy is conditioned or acquired and that these individuals are thus “decidedly approachable by psychotherapy” (Karpman, 1948, p. 458) and “a population for which early intervention or treatment in adulthood might be beneficial for society” (Porter, 1996, p. 187). This contrasts with the primary psychopath, whom Karpman (1948, p. 458) described as having “virtually nothing to work with psychotherapeutically.” In direct contradiction to Karpman’s latter claim, young children with CU traits show clinically meaningful and sustained improvements in behavioral and affective outcomes following treatment (Dadds et al., 2012, 2019; Fleming et al., 2022; Kimonis et al., 2019). However, without disaggregating children based on variant membership, these studies cannot address whether improved outcomes generalize across CU variants or if children with primary versus secondary CU traits have distinct treatment needs.

## The Present Study

Accordingly, the current study aims to investigate variant-specific patterns of treatment response among 3- to 7-year-old children with conduct problems and CU traits who participated in a targeted parenting program with their caregiver(s). We hypothesized that children classified as having secondary CU traits (i.e., elevated conduct problems, CU traits, and anxiety) would show significantly better treatment response and outcome in parent-rated child conduct problems, CU traits, affective empathy, and internalizing problems than children classified as having primary CU traits (i.e., elevated conduct problems and CU traits, and low to average anxiety). We also hypothesized that membership to the secondary CU traits group would be associated with significantly better treatment acceptability, including lower attrition and higher treatment satisfaction.

## Method

### Participants

Participants were 45 families of 3- to 7-year-old clinic-referred children (*M* age = 4.84 years, *SD* = 1.08, 84% boys) with conduct problems and CU traits, of whom *n* = 17 (38%) were classified as having clinically significant levels of anxiety. Participants were families who received a targeted intervention for CU-type conduct problems during one of two research trials: An open pilot trial that took place during May, 2014 to September, 2016 (*n* = 23; Kimonis et al., 2019) or a subsequent randomized controlled trial (RCT) that took place during January, 2016 to December, 2019 (*n* = 22; Fleming et al., 2022). Only data from participants randomized to the targeted intervention arm of the RCT were included in the current study. The RCT took place immediately following the open trial. The trials were identical in methodology, except for randomization in the RCT. Sample size was contingent on the number of families deemed eligible and who agreed to participate during the recruitment period for each trial. Participant recruitment and flow are published elsewhere (Kimonis et al., 2019; Fleming et al., 2022). See Table 1 for a description of the sample.

**Table 1:** Demographic Characteristics of Participants for the Overall Sample and By Primary and Secondary CU Classification Group

Variable	Overall Sample	Primary CU (Low Anxiety)	Secondary CU (High Anxiety)	
	*M* (*SD*)	*M* (*SD*)	*M* (*SD*)	Significance Test
Child age, years	*N* = 45 4.84 (1.08)	*n* = 28 5.07 (1.17)	*n* = 17 4.46 (0.80)	*t*(43) = 1.88, *p* = .07
Maternal age, years	*N* = 44 37.30 (5.55)	*n* = 27 37.63 (5.49)	*n* = 17 36.76 (5.77)	*U* = 235, *z* = 0.13, *p* = .89
Paternal age, years	*N* = 41 40.22 (6.06)	*n* = 27 40.11 (7.01)	*n* = 14 40.43 (3.82)	*U* = 220, *z* = 0.86, *p* = .41
	* N* (%)	*N* (%)	*N* (%)	
Child sex (parent-reported)	*N* = 45	*n* = 28	*n* = 17	Fisher’s exact test: *p* = .40
Male	38 (84.4)	25 (89.3)	13 (76.5)	
Female	7 (15.6)	3 (10.7)	4 (23.5)	
Maternal race/ethnicity	*N* = 43	*n* = 27	*n* = 16	Fisher’s exact test: *p* = .28
White	33 (76.7)	19 (70.4)	14 (87.5)	
Asian	6 (14.0)	5 (18.5)	1 (6.3)	
African	1 (2.3)	1 (3.7)	0 (0.0)	
Other race/ethnicity	3 (7.0)	2 (7.4)	1 (6.3)	
Paternal race/ethnicity	*N* = 42	*n* = 27	*n* = 15	Fisher’s exact test: *p* = .28
White	38 (90.5)	23 (85.2)	15 (100)	
Asian	3 (7.1)	3 (11.1)	0 (0.0)	
Pacific Islander	1 (2.4)	1 (3.7)	0 (0.0)	
Parent marital status	*N* = 43	*n* = 28	*n* = 15	Fisher’s exact test: *p* = .40
In relationship	37 (86.0)	23 (82.1)	14 (93.3)	
Not in relationship	6 (14.0)	5 (17.9)	1 (6.7)	
Annual household income	*N* = 38	*n* = 25	*n* = 13	χ^2^(1) = 0.32, *p* = .58
≤$150,000	21 (55.3)	13 (52.0)	8 (61.5)	
>$150,000	17 (44.7)	12 (48.0)	5 (38.5)	
	*M* (*SD*)	*M* (*SD*)	*M* (*SD*)	
	*N* = 45	*n* = 28	*n* = 17	
ECBI				
Intensity	176.73 (22.33)	168.25 (21.10)	190.69 (16.93)	*t*(43) = -3.72, *p* = < .001*, *d* = -1.14
Problem	24.47 (5.82)	22.50 (5.53)	27.71 (4.86)	*t*(43) = -3.20, *p* = .003*, *d* = -0.98
CBCL *T*-scores				
Aggressive Behavior	76.58 (11.01)	72.46 (9.28)	83.35 (10.46)	*t*(43) = -3.64, *p* = < .001*, *d* = -1.12
Oppositional Defiant	72.69 (7.18)	70.18 (7.39)	76.82 (4.52)	*U* = 373, *z* = 3.24, *p* = .001*, *d* = -1.03
Internalizing	64.60 (10.84)	58.82 (9.03)	74.12 (5.59)	*t*(42.96) = -6.28, *p* = < .001*, *d* = -1.93
ICU Total	37.06 (9.60)	35.82 (10.90)	39.09 (6.78)	*t*(43) = -1.11, *p* = .27, *d* = -0.34
GEM Affective	-4.78 (11.65)	-5.82 (13.04)	-3.06 (8.99)	*t*(43) = -0.77, *p* = .45, *d* = -0.25

*Note*. ECBI = Eyberg Child Behavior Inventory; CBCL = Child Behavior Checklist; ICU = Inventory of Callous-Unemotional Traits; GEM = Griffith Empathy Measure

*p < .05

Inclusion and exclusion criteria were identical across the two trials. Families were eligible to participate if they had a child between 3- and 7-years-old who (a) showed elevated CU traits on a 10-item version of the Inventory of Callous-Unemotional Traits (ICU) Preschool Version (Kimonis et al., 2016; see Appendix A) and (b) scored in the clinically significant range on at least one of the Achenbach System of Empirically Based Assessment (ASEBA; Achenbach & Rescorla, 2000, 2001) Child Behavior Checklist parent-reported externalizing-oriented scales. Families were ineligible if the participating caregiver(s) did not speak fluent English or if the child had received a primary mental health diagnosis other than Oppositional Defiant Disorder (ODD) or CD (e.g., severe autism spectrum), was deaf, or receiving concurrent psychological treatment for behavioral problems. Children with comorbidities such as Attention-Deficit/Hyperactivity Disorder (ADHD) and internalizing disorders were permitted to enroll if these problems were secondary to their conduct problems.

### Procedure

The trials were approved by University of New South Wales Human Research Ethics Committee (HC13234) and registered with the Australian New Zealand Clinical Trials Registry (ACTRN12616000280404). We obtained informed consent from all families before they completed a comprehensive in-person assessment taking approximately 2–2.5 h on five separate occasions: prior to treatment; following the first, second, and final treatment phases; and three months following treatment completion (see Supplemental Fig. 1). For each assessment, we invited each parent to complete a questionnaire battery via Qualtrics Survey Software. We attempted to conduct follow-up assessments with all families, regardless of dropout. However, of the *n* = 11 families who dropped out, five families failed to complete this assessment, while the remaining six families completed some (e.g., questionnaires only) or all components of the assessment. Assessors were not masked to assessment time point.

All families received Parent-Child Interaction Therapy (PCIT; McNeil and Hembree-Kigin, 2010) adapted for CU traits (PCIT-CU; Fleming and Kimonis, 2018). See Appendix A for an extended description of standard PCIT and PCIT-CU. Briefly, PCIT-CU differs from standard PCIT in three key ways: it (1) systematically and explicitly coaches parents to engage in warm, emotionally-responsive parenting during the first Child Directed Interaction (CDI) phase; (2) systematically augments punishment-based disciplinary strategies (i.e., time-out) with reward-based techniques (i.e., individualized token economy) during the second Parent Directed Interaction (PDI) phase; and (3) delivers a novel third phase called the Coaching And Rewarding Emotional Skills (CARES) module, which addresses the child’s insensitivity to distress cues via a variety of strategies (e.g., emotion recognition training; Fleming and Kimonis, 2018).

Using a fixed dosage approach, we delivered 21 one-hour intervention sessions for free to individual families at the UNSW Parent-Child Research Clinic, an urban university research clinic in Sydney, Australia. Families participated in one parent-only Teach session and six parent-child Coach sessions in each of the three PCIT-CU treatment phases (i.e., CDI-CU, PDI-CU, and CARES). We attempted to schedule weekly sessions; however, families were often unable to attend weekly (e.g., due to illness or vacation). Average length of treatment for completers was 35.74 (*SD* = 7.61, Range = 24.00–56.86) weeks. Average number of sessions completed for dropouts was 6.64 (*SD* = 5.39, Median = 8.00, Range = 0–15). One constant caregiver, typically the mother, completed all treatment/assessment sessions (see Appendix A for details of father involvement). Therapists (*n* = 10; 90% woman-identifying) were licensed, clinically-trained psychologists who received intensive in-vivo training from the last/senior author, who developed PCIT-CU and is a certified PCIT trainer, involving co-treatment roles on two cases and regular clinical supervision to maintain treatment fidelity. Therapists administered intervention to 4.50 cases on average (*SD* = 4.22, Range = 1–13).

### Measures

Appendix A provides details regarding the administration, scoring, and established psychometric properties of each measure. For classification and outcomes measures, we combined mother and father scores in a conservative fashion by taking the higher item-level rating between raters (i.e., ‘resolved’ score) to circumvent potential underreporting of problems at baseline and overreporting of treatment effects at post-treatment.

#### Eligibility Measures

One caregiver completed brief measures of CU traits and conduct problems during the initial intake telephone call to determine eligibility. Since it was important for this call to be brief, we used 10 items from the ICU (see Appendix A) to screen for CU traits. We selected these items because they reflect the four criteria of the DSM-5 LPE specifier. Parents rated children on a 4-point scale (0 = *Not at all true* to 3 = *Definitely true*). We considered CU traits ‘present’ when at least two of four LPE criteria were endorsed, as indicated by a ‘2’ or ‘3’ rating on its respective ICU items (Kimonis et al., 2015). In the current study, this method identified a sample with a mean baseline 24-item total ICU score of *M* = 37.06 (*SD* = 9.60), which fell over 1.5 standard deviation units above the mean for a community sample (*N* = 104) of 3- to 6-year-olds (Bansal et al., 2020). Conduct problems were considered ‘clinical’ if at least one of the externalizing-oriented scales on the ASEBA Child Behavior Checklist (CBCL) was in the clinical range (*T*-scores ≥ 70 or ≥ 64).

#### Classification Measure

Participants were classified as having clinically-significant anxiety problems if the DSM-Oriented Anxiety Problems on the appropriate age version of the ASEBA CBCL was in the clinical range (*T*-scores ≥ 70). In the current study, resolved scores demonstrated good internal consistency (CBCL1.5-5 and CBCL6-18: Cronbach’s αs = 0.82 and 0.75).

#### Outcome Measures

Participants completed the following measures at each of the five assessment points.

**Conduct problems.** We assessed child conduct problems using the Intensity and Problem scales of the Eyberg Child Behavior Inventory (ECBI; Eyberg and Pincus, 1999). Resolved Intensity and Problem scores showed good internal consistency (αs = 0.87 and 0.82). We also assessed conduct problems using resolved *T*-scores from the ASEBA CBCL1.5-5 and CBCL6-18 (Achenbach & Rescorla, 2000, 2001) Aggressive Behavior and DSM-Oriented Oppositional Defiant Problems. Resolved scores showed good internal consistency for the Aggressive Behavior scale (CBCL1.5-5 and CBCL6-18: αs = 0.87 and 0.82), acceptable internal consistency for the CBCL1.5-5 Oppositional Defiant Problems scale (α = 0.71), and low internal consistency for the CBCL6-18 Oppositional Defiant Problems scale (α = 0.58).

**CU traits.** We assessed CU traits using 24-item total scores from the preschool version of the ICU (Kimonis et al., 2016). Resolved total scores showed good internal consistency (α = 0.87).

**Affective empathy.** We assessed affective empathy using the affective empathy subscale from the Griffith Empathy Measure (GEM; Dadds et al., 2008). Resolved GEM Affective scores demonstrated good internal consistency (α = 0.87).

**Internalizing problems.** We assessed internalizing problems using resolved *T*-scores from the ASEBA CBCL1.5-5 and CBCL6-18 (Achenbach & Rescorla, 2000, 2001) Internalizing scale. Resolved scale scores demonstrated good to excellent internal consistency (CBCL1.5-5 and CBCL6-18: αs = 0.91 and 0.89).

#### Treatment Acceptability

We assessed attrition by recording the number of families who dropped out of treatment prior to completing all 21 PCIT-CU sessions. Following treatment, we assessed treatment satisfaction using the Therapy Attitude Inventory (TAI; Brestan et al., 1999). In the current study, total TAI scores demonstrated excellent internal consistency at post-CARES (α = 0.93) and good internal consistency at follow-up (α = 0.86).

### Planned Analyses

#### Baseline Differences

We conducted independent samples T-tests, Mann-Whitney U tests, chi-square tests of homogeneity, and Fisher’s exact test using SPSSv28 to evaluate baseline differences between Low (*n* = 28) and High (*n* = 17) Anxiety groups for demographic and parent-reported questionnaire variables. Regarding demographic variables, checks indicated outlier(s) and violations of normality for maternal and paternal age. Results of the non-parametric Mann-Whitney U test are thus reported in Table 1; however, when we re-ran T-tests with outlier(s) removed, the assumption of normality was no longer violated and direction and significance of results reflected those reported in Table 1. Regarding parent-reported questionnaire outcome variables, checks indicated outlier(s) for ECBI Intensity and CBCL Aggressive Behavior scales. Patterns of significance remained the same when outliers were removed. The assumption of normality was violated for CBCL Oppositional Defiant Problems, so results of non-parametric tests are reported in Table 1.

#### Outcome Measures

To examine variant differences in treatment outcomes, we conducted linear mixed-effects modeling (LMM) with the intent-to-treat sample (*N* = 45) using restricted maximum likelihood estimation to account for missing data. LMM was selected to account for the non-independence of data due to nesting of repeated observations (level 1, *n* = 183–186) within children (level 2, *N* = 45). All analyses were conducted in R version 4.2.0 (R Core Team, 2022) using the *lme4* and *lmerTest* packages. For each outcome variable, we first estimated a baseline random intercept model, following which we added the linear and quadratic effects of time (weeks since baseline), anxiety status, and the interactions of anxiety status with the linear and quadratic effects of time as fixed effects. For each outcome variable, we retained the model with the lowest model fit statistics (AIC and BIC) that also included the lowest-order time x anxiety status interaction effect since this was of *a priori* interest. We included sample (i.e., open trial vs. RCT) as a covariate in all models^1^. For each model, we specified two planned comparisons that examined group differences at post-treatment and follow-up time points. Fitted models were used to calculate estimated mean scores at each time point using the R package *emmeans*. Clinical significance was evaluated via between-subjects effects sizes (Cohen’s *d*) based on model-predicted means and pooled standard deviations. Assumptions were checked and satisfied for all LMM.

#### Treatment Acceptability

To examine group differences in attrition, we conducted binomial logistic regression using SPSSv28. We also evaluated baseline differences between completers (*n* = 34) and dropouts (*n* = 11) using a variety of tests. To examine group differences in treatment satisfaction, we conducted multiple linear regression using SPSSv28. We included sample as a covariate in all models. Assumptions were checked and satisfied.

## Results

### Baseline Differences

Table 1 presents descriptive information and analyses for demographic characteristics of participants for the full sample and by anxiety status. Regarding demographic variables, there were no significant differences between Low and High Anxiety groups in child age or sex, parent age, race/ethnicity (binarized: non-White/White), marital status (binarized: currently in/not in relationship), or household income (binarized using median income).

Regarding parent-reported questionnaire variables, as expected, children in the High Anxiety group were rated as having significantly higher baseline conduct problems than the Low Anxiety group on all variables. The anxiety groups did not differ on baseline CU traits or affective empathy. Unsurprisingly, the High Anxiety group had significantly higher baseline internalizing problems than the Low Anxiety group.

### Outcome Measures

**Fig. 1:**
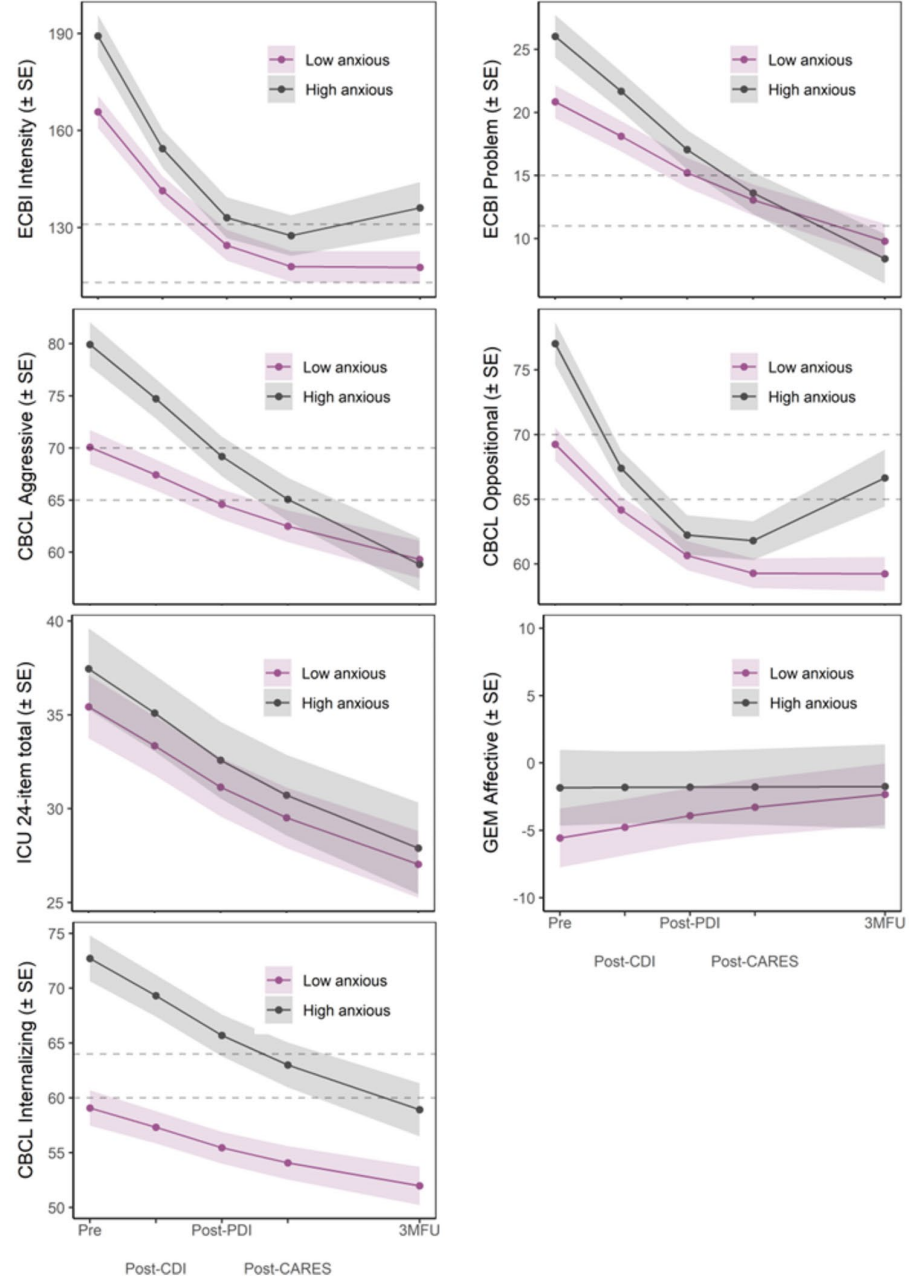
Plots of Model-Predicted Means *Note*. Plots of model-predicted means shown for primary outcomes at each assessment time point for Primary (Low Anxiety) and Secondary (High Anxiety). Dotted lines indicate borderline clinical and clinical ranges. ECBI = Eyberg Child Behavior Inventory; CBCL = Child Behavior Checklist; ICU = Inventory of Callous-Unemotional Traits; GEM = Griffith Empathy Measure; CDI = Child Directed Interaction; PDI = Parent Directed Interaction; CARES = Coaching and Rewarding Emotional Skills

Table 2 presents the results of the LMM, with additional details provided in Supplemental Tables 1–7. Figure 1 presents plots of model-predicted means (see Supplemental Tables 10–12 for observed resolved, mother-, and father-reported means).

**Table 2:** Results of Linear Mixed-Effects Models Examining the Linear and Quadratic Effects of Time, Anxiety Status, and Their Interactions on Outcomes

Estimates and *p* values										
Variable	Time		Time^2^		Anxiety		Time x Anxiety		Time^2^ x Anxiety	
	*b* [95% CI]	*p*	*b* [95% CI]	*p*	*b* [95% CI]	*p*	*b* [95% CI]	*p*	*b* [95% CI]	*p*
ECBI Intensity	-1.98 [-2.43 – -1.52]	< .001*	0.02 [0.01–0.03]	< .001*	23.47 [7.24–39.69]	.005*	-0.94 [-1.79 – -0.08]	.032*	0.01 [0.00–0.03]	.046*
ECBI Problem	-0.19 [-0.23 – -0.15]	< .001*	-	-	5.20 [0.97–9.42]	.016*	-0.11 [-0.19 – -0.03]	.005*	-	-
CBCL Agg	-0.18 [-0.25 – -0.12]	< .001*	-	-	9.84 [4.53–15.16]	< .001*	-0.18 [-0.29 – -0.06]	.002*	-	-
CBCL Opp Def	-0.41 [-0.57 – -0.26]	< .001*	0.00 [0.00–0.01]	.001*	7.78 [3.66–11.90]	< .001*	-0.42 [-0.70 – -0.13]	.004*	0.01 [0.00–0.01]	.004*
ICU 24-Item Total	-0.14 [-0.19 – -0.09]	< .001*	-	-	2.03 [-3.38–7.43]	.460	-0.02 [-0.11–0.07]	.654	-	-
GEM Affective	0.06 [-0.00–0.11]	.062	-	-	3.73 [-3.32–10.77]	.298	-0.05 [-0.16–0.05]	.312	-	-
CBCL Internalizing	-0.12 [-0.18 – -0.06]	< .001*	-	-	13.64 [8.44–18.84]	< .001*	-0.11 [-0.22 – -0.01]	.025*	-	-

*Note*. 95% CI = 95% confidence interval; Time = weeks since baseline; Anxiety = participant classification as Low vs. High anxiety; ECBI = Eyberg Child Behavior Inventory; CBCL = Child Behavior Checklist; Agg = Aggressive Behavior; Opp Def = Oppositional Defiant Problems; ICU = Inventory of Callous-Unemotional Traits; GEM = Griffith Empathy Measure; INT = Internalizing. Models control for sample and therapist. *p < .05

#### Conduct Problems

For the ECBI Intensity and CBCL Oppositional Defiant Problems scale scores, we found significant interactions of anxiety status with the linear and quadratic effects of time, indicating group differences in the rate and shape of change for these conduct problems outcomes. Figure 1 shows that the High Anxiety group had a faster rate of improvement relative to the Low Anxiety group during the active treatment period, but that these improvements deteriorated from post-treatment to follow-up for the High Anxiety group, with scores returning to the clinical or borderline clinical range at follow-up. In contrast, improvements in these outcomes maintained for the Low Anxiety group during the follow-up period, with scores remaining in the normal range at follow-up (*ds* = 0.75–1.11).

For the ECBI Problem and CBCL Aggressive Behavior scale scores, we found significant anxiety status by linear time interaction effects, indicating group differences in the rate of change over time for these conduct problems outcomes. Figure 1 shows that the High Anxiety group had a faster rate of change than the Low Anxiety group during the active treatment period. Both groups experienced maintenance of gains during follow-up, with scores remaining in the normal range for these scales at follow-up (*ds* = -0.22 – -0.06).

#### CU Traits

We found a significant effect of time but no significant anxiety status by time interaction effect for ICU scores, indicating that CU traits improved significantly over time but that rate of improvement did not significantly differ between Low and High Anxiety groups (Fig. 1). The group difference was not significant at follow-up (*d* = 0.11).

#### Affective Empathy

We did not find a significant effect of time nor significant anxiety status by time interaction effect for GEM Affective scale scores, indicating that affective empathy did not significantly improve over time for either anxiety group (Fig. 1). The group difference was not significant at follow-up (*d* = 0.06).

#### Internalizing Problems

We found a significant anxiety status by linear time interaction effect for CBCL Internalizing scale scores, indicating a group difference in the rate of change over time for internalizing symptoms. Figure 1 shows that the High Anxiety group had a faster rate of improvement than the Low Anxiety group. Both groups experienced maintenance during follow-up, with scores reaching or remaining in the normal range at follow-up (*d* = 0.87).

### Treatment Acceptability

Results of analyses are described, with full details provided in Supplemental Tables 8–9, including results of tests comparing treatment completers and dropouts (Supplemental Table 13). Completers and dropouts did not differ on any baseline variables, except for paternal race/ethnicity, such that the dropout group had a significantly higher proportion of fathers from a minoritized race or ethnicity (*n* = 3, 37.5%) compared to completers (*n* = 1, 2.9%) (Supplemental Table 13). Regarding anxiety status group differences, treatment retention was adequate in both Low Anxiety and High Anxiety groups, with 6 (21.4%) families in the Low Anxiety group and 5 (29.4%) families in the High Anxiety group dropping out of treatment. Supplemental Table 8 presents logistic regression results of group on treatment attrition, controlling for covariates. The model predicting treatment attrition was not significant, χ2(3) = 3.53, *p* = .32. Anxiety status did not predict attrition (*p* = .37).

Regarding treatment satisfaction reported by treatment completers for whom data were available, raw mean (*SD*) TAI scores for the Low Anxiety and High Anxiety groups, respectively, were 4.59 (0.47) and 4.63 (0.39) at post-CARES and 4.57 (0.43) and 4.67 (0.35) at three-month follow-up, which correspond to a high level of satisfaction with the process and outcome of therapy. Supplemental Table 9 presents separate multiple linear regression results of group on TAI scores at post-CARES and follow-up, controlling for covariates. The model predicting TAI scores was not significant at post-CARES, *F*(3,25) = 0.80, *p* = .51, R^2^ = 0.09, or follow-up, *F*(3,24) = 0.46, *p* = .72, R^2^ = 0.05. Anxiety status did not predict TAI scores at post-CARES (*p* = .73) or follow-up (*p* = .50).

## Discussion

The current study tested whether 3- to 7-year-old children with conduct problems and primary or secondary CU traits differentially responded to PCIT-CU. Consistent with hypotheses, we found that children classified as having secondary CU traits (i.e., clinically-significant conduct problems, CU traits, and anxiety) demonstrated a faster rate of change in parent-rated number of conduct problems and aggressive behavior symptoms than children classified as having primary CU traits (i.e., clinically-significant conduct problems and CU traits, and low to average anxiety), and that gains maintained over time for both groups. However, somewhat contrary to hypotheses, while children with secondary CU traits showed a faster rate of change in parent-rated frequency of conduct problems and oppositional defiant symptoms during the active treatment period, these gains deteriorated during the follow-up period. In contrast, children with primary CU traits maintained their gains during the follow-up period. Regarding affective outcomes, CU traits improved significantly and, contrary to our hypothesis, the rate of change in CU traits did not differ between primary and secondary variant groups. Unexpectedly, affective empathy did not improve significantly over time for either group. Consistent with our hypothesis, secondary CU traits were associated with a faster rate of improvement in internalizing problems than primary CU traits. Regarding treatment acceptability, there were no group differences in either treatment retention or satisfaction, which were high for both groups. We discuss these five key findings below.

Our first key finding is that all children showed improvement in CU traits, with no significant difference between the primary and secondary CU groups in rate of improvement. This finding suggests that PCIT-CU may adequately address the factors maintaining CU traits for both variants, despite their putative etiological differences. PCIT-CU was designed to address three key modifiable risk factors associated with primary CU traits: Low parental warmth, temperamentally-based punishment insensitivity, and *hypo*sensitivity to others’ distress cues (Fleming & Kimonis, 2018). For example, during the CARES phase, parent-child dyads are trained to recognize microexpressions associated with sadness and fear to improve their ability to attend to and recognize relevant socioemotional stimuli. In contrast, developmental models of secondary CU traits contend that temperamental *hyper*sensitivity increases the likelihood that repeated trauma exposure results in disengagement from emotional stimuli (Kimonis, 2023). It is possible that PCIT-CU’s CARES phase addresses a key maintaining factor implicated in this etiological account: Avoidance of emotional stimuli via disengagement. Consistent with treatment models for child anxiety, CARES may function as graded exposure to unpleasant emotional stimuli by systematically exposing children with secondary CU traits to increasingly realistic emotional displays or exchanges, starting with static photographs of emotional facial expressions and eventually progressing to role-plays and in-vivo coaching of emotional exchanges throughout the treatment phase. These proposed mechanisms of treatment-related change in CU traits should be formally tested in future research, including whether mechanisms differ based on CU variant. An important caveat to this discussion is that within-participant analyses of clinical improvement (Supplemental Table 14) indicated that 58% and 36% of treatment-completers from the primary and secondary groups, respectively, were rated below clinical cut-off on the ICU (< 31; Kimonis et al., 2014) at three-month follow-up. Thus, a substantial proportion of each anxiety group did not experience ‘normalization’ of CU traits. Critically, future mediation studies will also play a vital role in establishing whether putative mechanisms are being targeted effectively and, if not, how treatment targets for CU adaptations like PCIT-CU can be refined (Fleming, 2023).

Second, we found that some improvements in parent-reported conduct problems deteriorated for children with secondary CU traits. Specifically, deterioration occurred for scales assessing frequency of behaviors characterized by anger/irritability and emotional dysregulation (e.g., “temper tantrums or hot temper”) or non-compliance (“argues a lot”). Within-participant analyses indicated that 84% of treatment-completers in the primary CU group were rated in the normal range for oppositional defiant problems at follow-up, while 73% of completers in the secondary CU group were in the normal range (Supplemental Table 14). In contrast, scales for which children with secondary CU traits maintained gains tapped into aggression/rule-breaking (e.g., “physically attacks people”). Findings suggest that PCIT-CU may inadequately target factors that maintain anger/irritability, emotional dysregulation, and defiance when CU traits co-occur with anxiety. In standard PCIT, these problems are reduced via differential attention, such that parental attention is strategically withdrawn from angry, dysregulated, or defiant behaviors and returned when calm or compliant behavior occurs and/or time-out is completed (McNeil & Hembree-Kigin, 2010). While PCIT-CU retains these strategies, parents are also coached to attend to and reinforce children’s attention to, engagement with, and expression of emotions during the final CARES treatment phase. In this way, the CARES phase may muddy the water for families of children with secondary CU traits, among whom anger/irritability and dysregulation is more frequent than for children with primary CU traits (Kimonis, 2023). Specifically, parents may intermittently reinforce these behaviors when they apply CARES strategies and thus provide attention or allow escape from demands. Thus, families of children with secondary CU traits may require an adapted version of PCIT-CU that clearly demarcates times when parents should use strategies based on differential attention or to enhance emotional skills. Consistent with an existing adaptation of PCIT for children with ADHD (Chronis-Tuscano et al., 2016), PCIT-CU is likely to benefit from a strategy selection flowchart and *in-vivo* coaching of parents’ emotion skills-building strategies throughout all treatment phases. These strategies may reconcile the benefit CARES offers for targeting secondary CU traits, while remediating the risk of CARES strategies being applied inappropriately to address conduct problems.

An important research implication of this second key finding is that studies investigating the efficacy of treatment adaptations for children with CU-type conduct problems should consider CU variants in the design and analysis of clinical trials. To our knowledge, only four treatment outcome studies have recruited samples exclusively comprised of young children with clinically significant conduct problems and CU traits (Dadds et al., 2019; Fleming et al., 2022; Kimonis et al., 2019; White et al., 2022). None disaggregated the sample based on CU variant status. Given current findings, these studies may have underestimated treatment-related effects of CU adaptations for children with primary CU traits. Indeed, the findings of Fleming et al. (2022)—from which some current participants were drawn—suggested that PCIT-CU outperformed standard PCIT in improving rule-breaking behaviors but not behaviors characterized by anger/irritability, emotional dysregulation, or non-compliance. Although sample size precluded investigation of CU variants effects in this pilot RCT, future research should investigate if PCIT-CU and other treatment adaptations more uniformly outperform their standard versions specifically among children with primary CU traits, for whom the adaptations were developed.

Our third key finding is that PCIT-CU resulted in significant improvement in internalizing problems by follow-up for children with secondary CU traits, despite PCIT-CU not directly targeting this domain. Indeed, within-participant analyses indicated that only 33% off treatment-completers in the secondary CU group were rated in the normal range at post-treatment, but that this percentage increased to 73% at follow-up (Supplemental Table 14). Prior research also found that standard PCIT reduced comorbid internalizing symptoms, which was attributed to the generalizability of PCIT skills, such that parents can use the same strategies to reinforce both compliance and brave behavior (Chase & Eyberg, 2008). An alternative, albeit not mutually exclusive, explanation is that improvement in internalizing symptoms among children with secondary CU traits occurred because the discipline phase of PCIT-CU—during which parents are coached to implement a calm, fair, predictable time-out sequence incorporating an individualized token economy system—functioned as graded exposure to children’s trauma triggers. This is not a new idea: Some clinical researchers have argued that standard PCIT’s time-out sequence provides repeated exposure to safe, calm, and predictable limit-setting that extinguishes fear associated with trauma activators such as yelling and hitting (Quetsch et al., 2015). Given its discipline and reward system, it is possible that PCIT-CU addresses factors maintaining anxiety symptoms among children with CU traits who have experienced trauma, such as parental harshness, low parental warmth, and child emotional numbing. However, a key limitation of the current study is that we did not formally assess child trauma history or current symptoms. We were thus unable to establish or validate variant classification using prior trauma exposure nor specifically assess treatment-related change in trauma symptoms. It is critical that future research on PCIT-CU incorporates this trauma focus to explore these potential effects and their mechanisms.

Fourth, we did not find significant improvement in affective empathy following treatment for children with primary or secondary CU traits. This was surprising given improvement in CU traits, of which lack of empathy is a defining characteristic. However, both the reliability and validity of the measure we used to assess empathy have previously received criticism (Kimonis et al., 2021; Murphy, 2019). Although we attempted to circumvent some of these issues by using only the GEM Affective Empathy scale, which has stronger psychometric properties than its cognitive counterpart, the construct validity of the affective scale has also been questioned. Specifically, the affective scale failed to converge as expected with relevant constructs, leading Murphy (2019) to argue that its assessment of affective empathy is restricted to emotional contagion and may be more strongly related to negative emotionality than affective empathy. Kimonis et al. (2021) also argued that the GEM fails to assess empathy as a multidimensional construct, including dimensions that are particularly relevant to the development of empathy during the early childhood period (e.g., prosociality, sympathy). Thus, an important future direction for studies investigating variant-specific differences in treatment responsiveness is to investigate gains using a comprehensive, psychometrically-sound measure of empathy during the early childhood period, such as the Measure of Empathy in Early Childhood (MEEC; Chan et al., 2023; Kimonis et al., 2021).

Finally, there were no group differences in either treatment retention or satisfaction, which were generally high, demonstrating good engagement in PCIT-CU by families of children with both primary and secondary CU traits. While this is promising, it cannot be overlooked that the studies from which these data were drawn were conducted under ideal and highly controlled circumstances: Treatment was implemented at a university research clinic in a relatively affluent area of Sydney, Australia according to a comprehensive research protocol by practitioners trained and supervised by the intervention’s developer. Results thus require replication under real world conditions in an effectiveness trial with larger and more ethnically diverse samples of children. This is a critical next step in the dissemination and implementation of evidence-based treatment adaptations for children with CU-type conduct problems in community settings, which has been considerably lacking to date. While the issues of poor availability, accessibility, and acceptability of evidence-based treatment are not unique to children with CU-type conduct problems, we speculate that the relative newness of the ‘with limited prosocial emotions’ specifier for CD and persisting stigma attached to CU traits (Drent et al., 2022) compound these issues by reducing community practitioners’ awareness of and willingness to integrate this construct into their clinical practice, especially when children are young. In this way, efforts to reduce the research-to-practice gap via novel delivery methods such as video-teleconferencing (e.g., Fleming et al., 2017) or school delivery (e.g., Kyranides et al., 2018) will necessarily fall short if practitioners do not know about or are reluctant to engage with assessment tools and treatments specifically developed to target CU traits and their behavioral sequalae. Investigating community practitioner literacy and comfort regarding CU traits is an important avenue for future research.

Beyond the limitations already identified, which include absence of standardized assessment of historic and current trauma experiences and an affluent, homogenous sample, the current study has five other key limitations. First, our sample size was relatively small and likely underpowered to detect meaningful interaction effects. This study thus warrants replication with a larger and more diverse sample. Second, observed and teacher-reported changes in behavioral and affective outcomes were not examined, limiting the generalizability of findings across settings and informants. Third, families were only followed up to three months posttreatment, limiting generalizability over time. Fourth, we did not assess parent psychopathology, which has been implicated in etiological models of secondary CU traits (Meehan et al., 2017) and may thus be an important moderator or mediator of treatment-related change. Finally, we investigated the question of differential treatment responsiveness between CU variants to an intervention specifically developed to target primary CU-type conduct problems. While this is a critical research question, we also recommend future research investigate the extent to which comorbid anxiety impacts responsiveness to unadapted interventions, since this may be important to guiding clinical decision-making and represents a truer test of Karpman’s original assertion that secondary psychopathy is more amenable to treatment than primary psychopathy. It is also important that future studies include a no-treatment waitlist condition to eliminate the possibility of regression to the mean as an explanation of treatment effects.

Limitations notwithstanding, this is the first study to demonstrate that primary and secondary variants of young children with elevated CU traits may have different treatment needs. In a resounding challenge to Karpman’s assertion that primary psychopathy is not amenable to change, our findings showed that children with primary CU traits received significant and sustained benefit from an early intervention program tailored to address their unique needs. In contrast, interventions for children with secondary CU-type conduct problems may require further refinement to produce sustained gains across all outcomes, which should be the focus of future efficacy and effectiveness trials with longer-term follow-up periods. Further research is also required to establish the mechanisms by which interventions like PCIT-CU have their impact, including whether these mechanisms differ by CU variant, which will refine our understanding of key treatment targets needed to maximize change (Fleming, 2023). Finally, it is critical the field applies effort to disseminate and implement evidence-based treatment adaptations for CU-type conduct problems beyond the ivory tower. In this way, we echo recent calls for increased funding for research investigating the development, evaluation, and dissemination of evidence-based parenting programs (de Brito et al., 2021), extending this call by emphasising the need to fund research that specifically focuses on young children with the complex clinical profiles associated with both variants of CU traits.

### Electronic Supplementary Material

Below is the link to the electronic supplementary material.

Supplementary Material 1

Supplementary Material 2

## Footnotes

1 We were unable to control for the effect of therapist in the mixed-effects models due to model conversion failure. However, we have reported the results of ‘empty’ models specifying therapist as the single random factor to provide an indication of the percentage of variability explained by therapist for each outcome with the exception of the CBCL Internalizing Syndrome scale, which failed to converge (see Supplemental Tables 15–20).
