# Supplementary material for: Treatment Outcomes of Children with Primary Versus Secondary Callous-Unemotional Traits
Source: Res Child Adolesc Psychopathol. 2023 Aug 8;51(11):1581–94. doi: 10.1007/s10802-023-01112-6 (PMC10627936; doi:10.1007/s10802-023-01112-6)
Supplement: Supplementary file 1 — Supplementary Material 1 [file 10802_2023_1112_MOESM1_ESM.docx]

**Treatment outcomes of children with primary versus secondary callous-unemotional traits**

**Appendix A**

**Supplemental Method**

**Participants**

***Father Involvement in Treatment***

Of families who commenced treatment (*n* = 43), 84% of fathers participated in at least one treatment session. However, only 65% of fathers participated in > 25% of PCIT sessions, despite all primary caregivers being encouraged to attend given research finding father involvement enhances treatment effects (Tully et al., 2017).

**Procedure**

***Assessment Procedures***

See Supplemental Figure 1 for a visual representation of the study timeline.

***Treatment Procedures***

Standard PCIT comprises two distinct, sequential phases delivered via *in-vivo* coaching of parents using a wireless headset from behind a one-way mirror (McNeil & Hembree-Kigin, 2010). In standard PCIT, the first Child Directed Interaction (CDI) phase teaches parents a set of positive parenting skills to improve the parent-child relationship, including use of descriptive **P**raise, speech **R**eflections, behavior **I**mitation and **D**escription, and expressions of **E**njoyment. Known as the PRIDE or CDI ‘Do’ skills, parents are coached to use these strategies with the child within a play context. In PCIT-CU, CDI was adapted by replacing the ‘Enjoyment’ PRIDE skill with ‘**E**motional Expression’ to explicitly coach parents to increase their use of verbal (i.e., tone/pitch of voice, vocally expressed affection) and physical (i.e., touch, facial expressions) expressions of warmth (Fleming & Kimonis, 2018). In the second Parent Directed Interaction (PDI) phase of standard PCIT, parent(s) are coached to implement a consistent, predictable time-out procedure, used in response to child non-compliance or major rule violations (McNeil & Hembree-Kigin, 2010). In PCIT-CU, the PDI procedure was adapted by integrating an intensive reward-based behavior modification system involving the implementation of an individualized token economy system to motivate and reinforce positive child behaviors (e.g., compliance with commands and rules) (Fleming & KImonis, 2018). PCIT-CU augments standard PCIT with a novel third phase called **C**oaching **A**nd **R**ewarding **E**motional **S**kills (CARES), which addresses the child’s insensitivity to distress cues via a variety of strategies (e.g., emotion recognition training, positive reinforcement of prosocial and empathic behavior; Datyner et al., 2016; Kimonis & Armstrong, 2012). The key treatment objectives of CARES are to: (1) enhance attention to critical facial cues (i.e., micro-expressions) signaling distress in the self and others to improve emotion recognition and labeling; (2) improve emotional understanding by linking emotion to context, and identifying situations that trigger anger and frustration in the child; (3) teach prosocial and empathic behavior through therapist and parent modeling, role play, and social stories; (4) increase emotional labeling and prosocial behavior through positive reinforcement; and (5) increase child’s frustration tolerance through modeling, role-play, and reinforcing the child’s use of learned cognitive- behavioral strategies to decrease the incidence of aggressive behaviors (Fleming & Kimonis, 2018).

**Measures**

The following provides details regarding the administration, scoring, and established psychometric properties of each study measure.

***Eligibility Measures***

**Inventory of Callous-Unemotional Traits, 10-item version.** The Inventory of Callous-Unemotional Traits (ICU; Frick, 2004), Preschool version (Kimonis et al., 2016) is a 24-item parent-rated measure of callous-unemotional traits. To establish study eligibility during an initial intake telephone call, we used a 10-item version of the ICU. The items reflected the four criteria of the Limited Prosocial Emotions (LPE) specifier from the DSM-5. The following ICU items comprised the brief ICU measure, grouped by LPE criteria:

1. Lack of remorse or guilt: **Feels bad or guilty when he/she has done something wrong** (item 5), Easily admits to being wrong (item 13), Apologizes (“says he/she is sorry”) to persons he/she has hurt (item 16);
2. Callous-lack of empathy: **Is concerned about the feelings of others** (item 8), Tries not to hurt others’ feelings (item 17), Does things to make others feel good (item 24);
3. Lack of concern about performance: **Seems motivated to do his/her best in structured activities** (item 3), Always tries his/her best (item 15);
4. Shallow/deficient affect: Expresses his/her feelings openly (item 1), **Does not show emotions** (item 6, shallow/deficit affect).

Note that the bolded items were considered the primary indicator for each criterion. Parents rate their child’s behavior (e.g., “Does not show emotions”) on a 4-point scale (0 = not at all true, 1 = somewhat true, 2 = very true, 3 = definitely true). Scores range from 0 to 30, with higher scores indicating higher CU traits.

***Classification Measure***

**Achenbach System of Empirically Based Assessment Child Behavior Checklist– Preschool and School-Age Versions.** In the current study, parents completed the DSM-Oriented Anxiety Problems scale (e.g., “too fearful or anxious”) from the parent-rated ASEBA CBCL1.5-5 (Achenbach & Rescorla, 2000) and CBCL6-18 (Achenbach & Rescorla, 2001). Parents rated the applicability of each item on a 3-point scale from 0 (*not true*) to 2 (*very true or often true*), with scores summed to compute a total scale score. Total scores were then converted into *T­-*scores, which ranged from < 50–100. *T*-scores were considered ‘clinically significant’ if they were ≥ 70 (Achenbach & Rescorla, 2000, 2001). The DSM-oriented Anxiety Problems scale yielded a mean test-retest reliability coefficient of .80 over approximately 8 days, and a mean inter-parent reliability coefficient of .66 (Achenbach & Rescorla, 2000).

***Outcome Measures***

**Eyberg Child Behavior Inventory.** The Eyberg Child Behavior Inventory (ECBI; Eyberg & Pincus, 1999) is a 36-item parent-rated measure of child conduct problems. In the current study, parents rated the frequency of the child’s problem behaviors (e.g., “whines”) on a 7-point scale (from 1 “*never*” to 7 “*always*”), and indicated whether they currently considered the behavior problematic (*Yes/No*), with scores summed to compute Intensity and Problem Scales respectively. Intensity and Problem Scale scores range between 36–252 and 0–36, respectively, and raw scores were considered ‘clinically significant’ if they were ≥ 131 for the Intensity scale and ≥ 15 for the Problem scale. The Intensity and Problem scales have demonstrated excellent internal consistency (Cronbach’s αs =.95 and .93, Eyberg & Pincus, 1999), while the Intensity scale has demonstrated good inter-parent reliability (.69; Eisenstadt et al., 1994) and test-retest reliability across 12 weeks and 10 months (.80 and .75, respectively; Funderburk et al., 2003).

**Achenbach System of Empirically Based Assessment Child Behavior Checklist– Preschool and School-Age Versions, Aggressive Behavior and DSM-Oriented Oppositional Defiant Problems scales.** In the current study, parents completed selected externalizing-oriented scales from the parent-rated ASEBA CBCL1.5-5 (Achenbach & Rescorla, 2000) and CBCL6-18 (Achenbach & Rescorla, 2001), including the Aggressive Behavior (e.g., “hits others”) and DSM-Oriented Oppositional Defiant Problems (e.g., “defiant”) scales. Parents rated the applicability of each item on a 3-point scale from 0 (*not true*) to 2 (*very true or often true*), with scores summed to compute total scores for each scale. Total scores were then converted into *T­-*scores, which ranged from < 50 – 100. *T*-scores were considered ‘clinically significant’ if they were ≥ 70 (Achenbach & Rescorla, 2000, 2001). Aggressive Behavior and Oppositional Defiant Problems both yielded mean test-retest reliability coefficients of .87 over approximately 1 to 2 weeks, respectively, and mean inter-parent reliability coefficients of .66 and .65, respectively (Achenbach & Rescorla, 2000).

**Inventory of Callous-Unemotional Traits.** The Inventory of Callous-Unemotional Traits (ICU; Frick, 2004), Preschool version (Kimonis et al., 2016) is a 24-item parent-rated measure of callous-unemotional traits (e.g., “Feels bad or guilty when he/she has done something wrong”). The Preschool version of the ICU was adapted from the original Parent version to ensure the measure was developmentally appropriate for preschool-age children. Specifically, the original Item 3 (i.e., “Is concerned about schoolwork”) was replaced with “Seems motivated to do his/her best in structured activities.” Total ICU scores range from 0–72 and have demonstrated acceptable internal consistency and expected correlations with criterion measures such as reduced emotional responding to distress cues and severe aggression, across a wide age range, sex, types of samples, and different language translations (e.g., Ezpeleta et al., 2013; Kimonis et al., 2016). Preschool children rated high on the ICU by parents and teachers were more likely to be antisocial and aggressive, score high on other psychopathy dimensions, and show emotional attention and recognition impairments than low-scoring children (Kimonis et al., 2016).

**Griffith Empathy Measure.** The Griffith Empathy Measure (GEM; Dadds et al., 2008) is a 23-item parent-rated measure of empathy, with items assessing cognitive (e.g., “my child has trouble understanding other people’s feelings”) and affective (e.g., “seeing another child sad makes my child feel sad”) indicators of empathy. In the current study, only the 9-item Affective Empathy scale was used, due to concerns regarding the Cognitive Empathy scale’s psychometric properties and developmental appropriateness for young children (see Kimonis et al., 2021). Parents rated each item on a 9-point Likert scale from *strongly disagree* (-4) to *strongly agree* (+4), with scores summed to create an Affective Empathy score ranging from -36–36. Prior studies have demonstrated good test-retest reliability of affective empathy scores over 1 week (*r* = .89), good internal consistency, and acceptable inter-parental agreement (*r* > .41) (Dadds et al., 2008).

**Achenbach System of Empirically Based Assessment Child Behavior Checklist– Preschool and School-Age Versions, Internalizing scale.** In the current study, parents completed the Internalizing composite scale (e.g., “Unhappy, sad, or depressed,” “Nervous, highstrung, or tense”) from the parent-rated ASEBA CBCL1.5-5 (Achenbach & Rescorla, 2000) and CBCL6-18 (Achenbach & Rescorla, 2001). Parents rated the applicability of each item on a 3-point scale from 0 (*not true*) to 2 (*very true or often true*), with scores summed to compute a total scale score. Total scores were then converted into *T­-*scores, which ranged from < 50 – 100. *T*-scores were considered ‘clinically significant’ if they were ≥ 64 (Achenbach & Rescorla, 2000, 2001). The Internalizing scale yielded a mean test-retest reliability coefficient of .90 over approximately 1 to 2 weeks, and a mean inter-parent reliability coefficient of .59 (Achenbach & Rescorla, 2000).

**Therapy Attitude Inventory.** The Therapy Attitude Inventory (TAI; Brestan et al., 1999) is a 10-item parent-rated measure of level of satisfaction with the process and outcome of therapy. In the current study, parents rated each item on a scale from 1 (*dissatisfaction with treatment* or *worsening of problems*) to 5 *(maximum satisfaction with treatment* or *improvement of problems*). TAI total scores demonstrated excellent internal consistency (α = .91) and test-retest reliability (*r* = .85) across four months from post-treatment to follow-up assessments (Brestan et al., 1999).

**Supplemental Figure 1**

*Study Timelines for PCIT-CU Open Trial (A) and PCIT-CU RCT (B)*

*
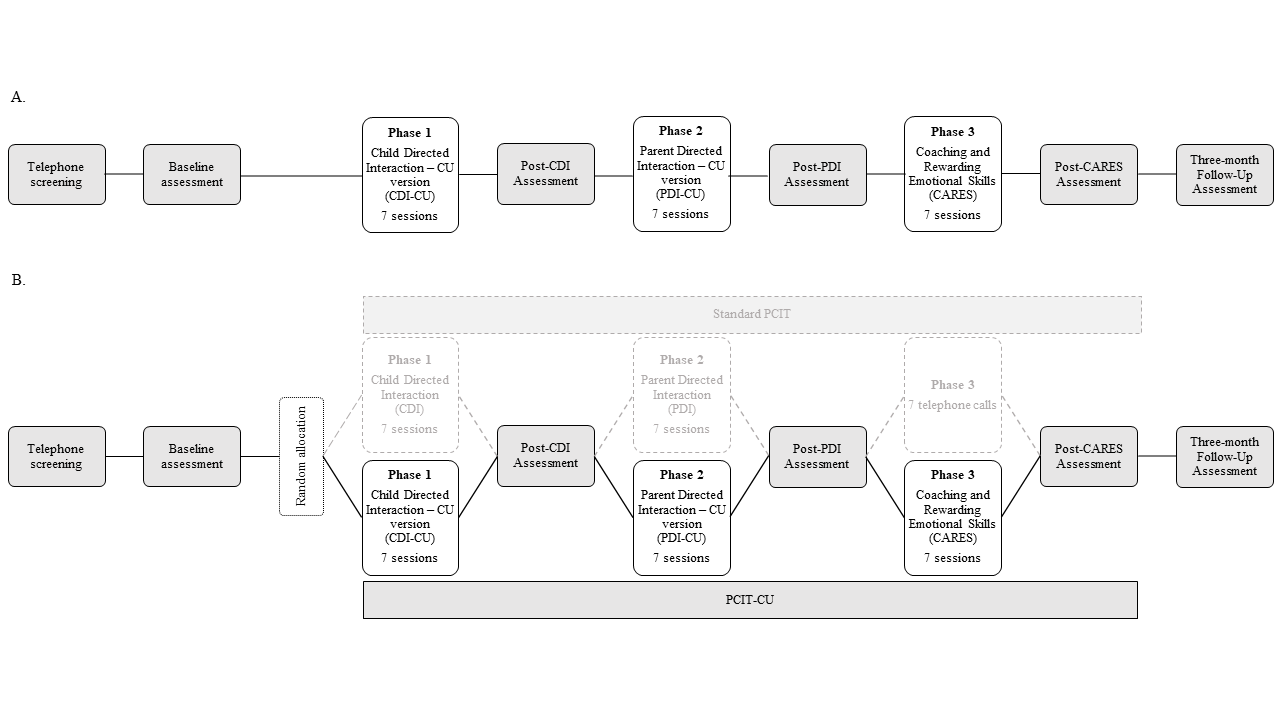
*

*Note.* Figure depicts the study timelines for the PCIT-CU Open Trial (Supplemental Figure 1A) and PCIT-CU Randomized Controlled Trial (RCT) (Supplemental Figure 1B). Note that data from participants randomly allocated to the Standard PCIT treatment arm in the RCT (indicated by dashed lines) were not included in the current study. Study procedures were identical across the two studies, except for randomization in the RCT. The average number of weeks between assessment points are baseline to post-CDI: 14.39 (*SD* = 5.91); post-CDI to post-PDI: 15.39 (*SD* = 6.30); post-PDI to post-CARES: 12.38 (*SD* = 5.20); post-CARES to three-month follow-up: 17.55 (*SD* = 8.90). CDI = Child Directed Interaction phase; PDI = Parent Directed Interaction phase; CARES = Coaching and Rewarding Emotional Skills module; CU = callous-unemotional.

**References**

Achenbach, T. M., & Rescorla, L. A. (2000; 2001). *Manual for the ASEBA Preschool/ School-Age Forms & Profiles*. University of Vermont Research Center for Children, Youth, & Families.

Brestan, E. V., Jacobs, J. R., Rayfield, A. D., & Eyberg, S. M. (1999). A consumer satisfaction measure for parent-child treatments and its relation to measures of child behavior change. *Behavior Therapy, 30*, 17-30. <https://doi.org/10.1016/S0005-7894(99)80043-4>

Dadds, M. R., Hunter, K., Hawes, D. J., Frost, A. D., Vassallo, S., Bunn, P., Merz, S., & El Masry, Y. (2008). A measure of cognitive and affective empathy in children using parent ratings. *Child Psychiatry & Human Development, 39*, 111-122. <https://doi.org/10.1007/s10578-007-0075-4>

Datyner, A., Kimonis, E. R., Hunt, E., & Armstrong, K. (2016). Using a novel emotional skills module to enhance empathic responding for a child with conduct disorder with limited prosocial emotions. *Clinical Case Studies, 15*(1), 35-52. <https://doi.org/10.1177/1534650115588978>

Eisenstadt, T. H., McElreath, L. H., Eyberg, S. M., & McNeil, C. B. (1994). Interparent agreement on the Eyberg Child Behavior Inventory. *Child and Family Behavior Therapy, 16*(1), 21-28. <https://doi.org/10.1300/J019v16n01_02>

Eyberg S. M., & Pincus D. (1999). *Eyberg Child Behavior Inventory and Sutter-Eyberg Student Behavior Inventory—Revised: Professional manual.*  Psychological Assessment Resources.

Ezpeleta, L., Osa, N. D. L., Granero, R., Penelo, E., & Domènech, J. M. (2013). Inventory of callous-unemotional traits in a community sample of preschoolers. *Journal of Clinical Child and Adolescent Psychology*, *42*(1), 91-105. <https://doi.org/10.1080/15374416.2012.734221>

Fleming, G. E., & Kimonis, E. R. (2018). PCIT for Children with Callous-Unemotional Traits. In L. N. Niec (Ed.), *Handbook of Parent-Child Interaction Therapy: Innovations and applications for research and practice* (pp. 19-34). Springer.

Frick, P. J. (2004). *The Inventory of Callous-Unemotional Traits*. University of New Orleans.

Funderburk, B. W., Eyberg, S. M., Rich, B. A., & Behar, L. (2003). Further psychometric evaluation of the Eyberg and Behar rating scales for parents and teachers of preschoolers. *Early Education and Development, 14*(1), 67-82. <https://doi.org/10.1207/s15566935eed1401_5>

Kimonis, E. R., & Armstrong, K. (2012). Adapting parent–child interaction therapy to treat severe conduct problems with callous-unemotional traits: A case study. *Clinical Case Studies*, *11*(3), 234-252. <https://doi.org/10.1177/1534650112448835>

Kimonis, E. R., Fanti, K. A., Anastassiou-Hadjicharalambous, X., Mertan, B., Goulter, N., & Katsimicha, E. (2016). Can callous-unemotional traits be reliably measured in preschoolers? *Journal of Abnormal Child Psychology*, *44*(4), 625-638. <https://doi.org/10.1007/s10802-015-0075-y>

Kimonis, E. R., Fanti, K., Goldweber, A., Marsee, M. A., Frick, P. J., & Cauffman, E. (2014). Callous-unemotional traits in incarcerated adolescents. *Psychological Assessment*, *26*(1), 227-237. <https://doi.org/10.1037/a0034585>

Kimonis, E. R., Jain, N., Neo, B., Fleming, G. E., & Briggs, N. (2021). Development of an empathy rating scale for young children. *Assessment, 30*(1), 37–50. <https://doi.org/10.1177/10731911211038629>

McNeil, C., & Hembree-Kigin, T. L. (2010). *Parent-Child Interaction Therapy*. Springer.

Tully, L. A., Piotrowska, P. J., Collins, D. A. J., Mairet, K. S., Black, N., Kimonis, E. R., Hawes, D. J., Moul, C., Lenroot, R. K., Frick, P. J, Anderson,V., & Dadds, M. R. (2017). Optimizing child outcomes from parenting interventions: Fathers' experiences, preferences and barriers to participation. *BMC Public Health, 17*, 550. <https://doi.org/10.1186/s12889-017-4426-1>
