# Supplementary material for: Treatment Outcomes of Children with Primary Versus Secondary Callous-Unemotional Traits
Source: Res Child Adolesc Psychopathol. 2023 Aug 8;51(11):1581–94. doi: 10.1007/s10802-023-01112-6 (PMC10627936; doi:10.1007/s10802-023-01112-6)
Supplement: Supplementary file 2 — Supplementary Material 2 [file 10802_2023_1112_MOESM2_ESM.docx]

**Treatment outcomes of children with primary versus secondary callous-unemotional traits**

**Appendix B**

**Supplemental Results**

**Supplemental Table 1**

*Linear Mixed Model Results for Resolved Eyberg Child Behavior Inventory (ECBI) Intensity Raw Scores*

|  | Estimate (*SE*) | 95% CI | *p* |  |  |
| --- | --- | --- | --- | --- | --- |
| **Fixed Effects** | | | | |  |
| Intercept | 159.32 | 136.21 – 182.44 | **<0.001** |  |  |
| Time | -1.98 | -2.43 – -1.52 | **<0.001** |  |  |
| Anxiety status | 23.47 | 7.24 – 39.69 | **0.005** |  |  |
| Time*Time | 0.02 | 0.01 – 0.03 | **<0.001** |  |  |
| Sample | 4.30 | -9.36 – 17.96 | 0.535 |  |  |
| Time*Anxiety status | -0.94 | -1.79 – -0.08 | **0.032** |  |  |
| Time*Time*Anxiety status | 0.01 | 0.00 – 0.03 | **0.046** |  |  |
| **Random Effects** | | | | |  |
| σ^2^ | 320.45 |  |  |  |  |
| τ_00,ID_ | 436.70 |  |  |  |  |
| ICC | 0.58 |  |  |  |  |
| N_ID_ | 45 |  |  |  |  |
| Observations | 186 |  |  |  |  |
| Marginal R^2^ / Conditional R^2^ | 0.396 / 0.744 |  |  |  |  |
| **Testing Group Differences in Model-Predicted Scores** | | | | |  |
|  | Estimate (*SE*) | *df* | *t* | *p* | *d* |
| Post-CARES | -9.59 (7.89) | 58.2 | -1.215 | 0.2292 | 0.439 |
| Primary (High Anx): | *M* = 117.93, *SD* = 21.96 | |  |  |  |
| Secondary (Low Anx): | *M* = 127.52, *SD* = 21.69 | |  |  |  |
| 3MFU | -18.43 (9.40) | 103.0 | -1.960 | 0.0527 | 0.745 |
| Primary (High Anx): | *M =* 117.70, *SD* = 23.12 | |  |  |  |
| Secondary (Low Anx): | *M* = 136.13, *SD* = 26.27 | |  |  |  |

*Note*. *SE* = standard error; 95% CI = 95% confidence interval; Time = weeks since baseline; Sample = participant classification as Open Trial vs. RCT; Anxiety status = participant classification as Low vs. High anxiety; CARES = Coaching and Rewarding Emotional Skills; 3MFU = three month follow-up assessment; Anx = Anxiety.

**Supplemental Table 2**

*Linear Mixed Model Results for Resolved Eyberg Child Behavior Inventory (ECBI) Problem Raw Scores*

|  | Estimate (*SE*) | 95% CI | *p* |  |  |
| --- | --- | --- | --- | --- | --- |
| **Fixed Effects** | | | | |  |
| Intercept | 15.99 | 9.82 – 22.17 | **<0.001** |  |  |
| Time | -0.19 | -0.23 – -0.15 | **<0.001** |  |  |
| Anxiety status | 5.20 | 0.97 – 9.42 | **0.016** |  |  |
| Sample | 3.23 | -0.47 – 6.94 | 0.087 |  |  |
| Time*Anxiety status | -0.11 | -0.19 – -0.03 | **0.005** |  |  |
| **Random Effects** | | | | |  |
| σ^2^ | 26.54 |  |  |  |  |
| τ_00,ID_ | 31.42 |  |  |  |  |
| ICC | 0.54 |  |  |  |  |
| N_ID_ | 45 |  |  |  |  |
| Observations | 186 |  |  |  |  |
| Marginal R^2^ / Conditional R^2^ | 0.326 / 0.692 |  |  |  |  |
| **Testing Group Differences in Model-Predicted Scores** | | | | |  |
|  | Estimate (*SE*) | *df* | *t* | *p* | *d* |
| Post-CARES | -0.547 (2.07) | 53.6 | -0.264 | 0.7928 | 0.096 |
| Primary (High Anx): | *M* = 13.06, *SD* = 5.63 | |  |  |  |
| Secondary (Low Anx): | *M* = 13.61, *SD* = 5.77 | |  |  |  |
| 3MFU | 1.405 (2.41) | 86.3 | 0.584 | 0.5609 | -0.218 |
| Primary (High Anx): | *M* = 9.79, *SD* = 6.34 | |  |  |  |
| Secondary (Low Anx): | *M* = 8.39, *SD* = 6.52 | |  |  |  |

*Note*. *SE* = standard error; 95% CI = 95% confidence interval; Time = weeks since baseline; Sample = participant classification as Open Trial vs. RCT; Anxiety status = participant classification as Low vs. High anxiety; CARES = Coaching and Rewarding Emotional Skills; 3MFU = three month follow-up assessment; Anx = Anxiety.

**Supplemental Table 3**

*Linear Mixed Model Results for Resolved Child Behavior Checklist (CBCL) Aggressive Behavior T-scores*

|  | Estimate (*SE*) | 95% CI | *p* |  |  |
| --- | --- | --- | --- | --- | --- |
| **Fixed Effects** | | | | |  |
| Intercept | 66.07 | 58.54 – 73.60 | **<0.001** |  |  |
| Time | -0.18 | -0.25 – -0.12 | **<0.001** |  |  |
| Anxiety status | 9.84 | 4.53 – 15.16 | **<0.001** |  |  |
| Sample | 2.67 | -1.82 – 7.17 | 0.242 |  |  |
| Time*Anxiety status | -0.18 | -0.29 – -0.06 | **0.002** |  |  |
| **Random Effects** | | | | |  |
| σ^2^ | 53.81 |  |  |  |  |
| τ_00,ID_ | 42.29 |  |  |  |  |
| ICC | 0.44 |  |  |  |  |
| N_ID_ | 45 |  |  |  |  |
| Observations | 184 |  |  |  |  |
| Marginal R^2^ / Conditional R^2^ | 0.287 / 0.601 |  |  |  |  |
| **Testing Group Differences in Model-Predicted Scores** | | | | |  |
|  | Estimate (*SE*) | *df* | *t* | *p* | *d* |
| Post-CARES | -2.576 (2.57) | 53.0 | -1.001 | 0.3215 | 0.364 |
| Primary (High Anx): | *M* = 62.50, *SD* = 6.99 | |  |  |  |
| Secondary (Low Anx): | *M* = 65.07, *SD* = 7.17 | |  |  |  |
| 3MFU | 0.475 (3.11) | 94.9 | 0.153 | 0.8790 | -0.057 |
| Primary (High Anx): | *M* = 59.31, *SD* = 8.23 | |  |  |  |
| Secondary (Low Anx): | *M* = 58.84, *SD* = 8.42 | |  |  |  |

*Note*. *SE* = standard error; 95% CI = 95% confidence interval; Time = weeks since baseline; Sample = participant classification as Open Trial vs. RCT; Anxiety status = participant classification as Low vs. High anxiety; CARES = Coaching and Rewarding Emotional Skills; 3MFU = three month follow-up assessment; Anx = Anxiety.

**Supplemental Table 4**

*Linear Mixed Model Results for Resolved Child Behavior Checklist (CBCL) DSM-Oriented Oppositional Defiant Problems T-scores*

|  | Estimate (*SE*) | 95% CI | *p* |  |  |
| --- | --- | --- | --- | --- | --- |
| **Fixed Effects** | | | | |  |
| Intercept | 63.46 | 58.28 – 68.63 | **<0.001** |  |  |
| Time | -0.41 | -0.57 – -0.26 | **<0.001** |  |  |
| Anxiety status | 7.78 | 3.66 – 11.90 | **<0.001** |  |  |
| Time*Time | 0.00 | 0.00 – 0.01 | **0.001** |  |  |
| Sample | 3.86 | 0.89 – 6.83 | **0.011** |  |  |
| Time*Anxiety status | -0.42 | -0.70 – -0.13 | **0.004** |  |  |
| Time*Time*Anxiety status | 0.01 | 0.00 – 0.01 | **0.004** |  |  |
| **Random Effects** | | | | |  |
| σ^2^ | 36.17 |  |  |  |  |
| τ_00,ID_ | 15.24 |  |  |  |  |
| ICC | 0.30 |  |  |  |  |
| N_ID_ | 45 |  |  |  |  |
| Observations | 184 |  |  |  |  |
| Marginal R^2^ / Conditional R^2^ | 0.376 / 0.561 |  |  |  |  |
| **Testing Group Differences in Model-Predicted Scores** | | | | |  |
|  | Estimate (*SE*) | *df* | *t* | *p* | *d* |
| Post-CARES | -2.51 (1.88) | 74.8 | -1.335 | 0.1858 | 0.483 |
| Primary (High Anx): | *M* = 59.29, *SD* = 5.22 | |  |  |  |
| Secondary (Low Anx): | *M* = 61.80, *SD* = 5.19 | |  |  |  |
| 3MFU | -7.41 (2.57) | 149.4 | -2.887 | **0.0045** | 1.11 |
| Primary (High Anx): | *M* = 59.24, *SD* = 6.02 | |  |  |  |
| Secondary (Low Anx): | *M* = 66.65, *SD* = 7.31 | |  |  |  |

*Note*. *SE* = standard error; 95% CI = 95% confidence interval; Time = weeks since baseline; Sample = participant classification as Open Trial vs. RCT; Anxiety status = participant classification as Low vs. High anxiety; CARES = Coaching and Rewarding Emotional Skills; 3MFU = three month follow-up assessment; Anx = Anxiety.

**Supplemental Table 5**

*Linear Mixed Model Results for Resolved Inventory of Callous-Unemotional Traits (ICU) Total Raw Scores*

|  | Estimate (*SE*) | 95% CI | *p* |  |  |
| --- | --- | --- | --- | --- | --- |
| **Fixed Effects** | | | | |  |
| Intercept | 39.97 | 31.82 – 48.11 | **<0.001** |  |  |
| Time | -0.14 | -0.19 – -0.09 | **<0.001** |  |  |
| Anxiety status | 2.03 | -3.38 – 7.43 | 0.460 |  |  |
| Sample | -3.03 | -7.92 – 1.86 | 0.223 |  |  |
| Time*Anxiety status | -0.02 | -0.11 – 0.07 | 0.654 |  |  |
| **Random Effects** | | | | |  |
| σ^2^ | 31.33 |  |  |  |  |
| τ_00,ID_ | 58.69 |  |  |  |  |
| ICC | 0.65 |  |  |  |  |
| N_ID_ | 45 |  |  |  |  |
| Observations | 183 |  |  |  |  |
| Marginal R^2^ / Conditional R^2^ | 0.136 / 0.699 |  |  |  |  |
| **Testing Group Differences in Model-Predicted Scores** | | | | |  |
|  | Estimate (*SE*) | *df* | *t* | *p* | *d* |
| Post-CARES | -1.207 (2.69) | 47.0 | -0.448 | 0.6561 | 0.163 |
| Primary (High Anx): | *M* = 29.51, *SD* = 7.40 | |  |  |  |
| Secondary (Low Anx): | *M* = 30.72, *SD* = 7.45 | |  |  |  |
| 3MFU | -0.863 (3.02) | 70.6 | -0.285 | 0.7762 | 0.106 |
| Primary (High Anx): | *M* = 27.03, *SD* = 8.14 | |  |  |  |
| Secondary (Low Anx): | *M* = 27.89, *SD* = 8.10 | |  |  |  |

*Note*. *SE* = standard error; 95% CI = 95% confidence interval; Time = weeks since baseline; Sample = participant classification as Open Trial vs. RCT; Anxiety status = participant classification as Low vs. High anxiety; CARES = Coaching and Rewarding Emotional Skills; 3MFU = three month follow-up assessment; Anx = Anxiety.

**Supplemental Table 6**

*Linear Mixed Model Results for Resolved Griffith Empathy Measure (GEM) Affective Empathy Raw Scores*

|  | Estimate (*SE*) | 95% CI | *p* |  |  |
| --- | --- | --- | --- | --- | --- |
| **Fixed Effects** | | | | |  |
| Intercept | -11.51 | -22.24 – -0.79 | **0.036** |  |  |
| Time | 0.06 | -0.00 – 0.11 | 0.062 |  |  |
| Anxiety status | 3.73 | -3.32 – 10.77 | 0.298 |  |  |
| Sample | 3.97 | -2.48 – 10.41 | 0.226 |  |  |
| Time*Anxiety status | -0.05 | -0.16 – 0.05 | 0.312 |  |  |
| **Random Effects** | | | | |  |
| σ^2^ | 45.66 |  |  |  |  |
| τ_00,ID_ | 104.60 |  |  |  |  |
| ICC | 0.70 |  |  |  |  |
| N_ID_ | 45 |  |  |  |  |
| Observations | 185 |  |  |  |  |
| Marginal R^2^ / Conditional R^2^ | 0.040 / 0.708 |  |  |  |  |
| **Testing Group Differences in Model-Predicted Scores** | | | | |  |
|  | Estimate (*SE*) | *df* | *t* | *p* | *d* |
| Post-CARES | -1.513 (3.52) | 47.3 | -0.430 | 0.6691 | 0.156 |
| Primary (High Anx): | *M* = -3.28, *SD* = 9.68 | |  |  |  |
| Secondary (Low Anx): | *M* = -1.77, *SD* = 9.71 | |  |  |  |
| 3MFU | -0.584 (3.88) | 67.1 | -0.150 | 0.8809 | 0.0560 |
| Primary (High Anx): | *M* = -2.32, *SD* = 10.47 | |  |  |  |
| Secondary (Low Anx): | *M* - -1.74, *SD* = 10.38 | |  |  |  |

*Note*. *SE* = standard error; 95% CI = 95% confidence interval; Time = weeks since baseline; Sample = participant classification as Open Trial vs. RCT; Anxiety status = participant classification as Low vs. High anxiety; CARES = Coaching and Rewarding Emotional Skills; 3MFU = three month follow-up assessment; Anx = Anxiety.

**Supplemental Table 7**

*Linear Mixed Model Results for Resolved Child Behavior Checklist (CBCL) Internalizing Syndrome T-scores*

|  | Estimate (*SE*) | 95% CI | *p* |  |  |
| --- | --- | --- | --- | --- | --- |
| **Fixed Effects** | | | | |  |
| Intercept | 56.99 | 49.42 – 64.57 | **<0.001** |  |  |
| Time | -0.12 | -0.18 – -0.06 | **<0.001** |  |  |
| Anxiety status | 13.64 | 8.44 – 18.84 | **<0.001** |  |  |
| Sample | 1.39 | -3.14 – 5.92 | 0.546 |  |  |
| Time*Anxiety status | -0.11 | -0.22 – -0.01 | **0.025** |  |  |
| **Random Effects** | | | | |  |
| σ^2^ | 41.71 |  |  |  |  |
| τ_00,ID_ | 46.42 |  |  |  |  |
| ICC | 0.53 |  |  |  |  |
| N_ID_ | 45 |  |  |  |  |
| Observations | 184 |  |  |  |  |
| Marginal R^2^ / Conditional R^2^ | 0.324 / 0.680 |  |  |  |  |
| **Testing Group Differences in Model-Predicted Scores** | | | | |  |
|  | Estimate (*SE*) | *df* | *t* | *p* | *d* |
| Post-CARES | -8.93 (2.55) | 52.2 | -3.500 | **0.0010** | 1.27 |
| Primary (High Anx): | *M* = 54.07, *SD* = 6.96 | |  |  |  |
| Secondary (Low Anx): | *M* = 63.00, *SD* = 7.08 | |  |  |  |
| 3MFU | -6.95 (2.99) | 86.7 | -2.326 | **0.0224** | 0.868 |
| Primary (High Anx): | *M* = 51.97, *SD* = 7.97 | |  |  |  |
| Secondary (Low Anx): | *M* = 58.92, *SD* = 8.04 | |  |  |  |

*Note*. *SE* = standard error; 95% CI = 95% confidence interval; Time = weeks since baseline; Sample = participant classification as Open Trial vs. RCT; Anxiety status = participant classification as Low vs. High anxiety; CARES = Coaching and Rewarding Emotional Skills; 3MFU = three month follow-up assessment; Anx = Anxiety.

**Supplemental Table 8**

*Results of Logistic Regression Analyses Examining the Effects of Anxiety Status Classification on Treatment Attrition*

|  | *b* (*SE*) | Wald^2^ | *df* | *p* | Odds Ratio | 95% CI for Odds Ratio | |
| --- | --- | --- | --- | --- | --- | --- | --- |
|  |  |  |  |  |  | Lower | Upper |
| Sample | 1.63 (1.15) | 2.00 | 1 | .16 | 5.12 | 0.53 | 48.90 |
| Therapist | -0.42 (0.24) | 3.01 | 1 | .08 | 0.66 | 0.41 | 1.06 |
| Anxiety status | -0.68 (0.76) | 0.79 | 1 | .37 | 0.51 | 0.11 | 2.26 |

*Note*. *SE* = standard error; 95% CI = 95% confidence interval; Post = post-CARES assessment; 3MFU = three-month follow-up assessment; Sample = participant classification as Open Trial vs. RCT; Anxiety status = participant classification as Low vs. High anxiety.

**Supplemental Table 9**

*Results of Multiple Regression Analyses Examining the Effect of Anxiety Status Classification on Resolved Treatment Satisfaction Scores*

|  | *b* (*SE*) | β | *p* |
| --- | --- | --- | --- |
| Post |  |  |  |
| Sample | -0.42 (0.28) | -0.49 | .15 |
| Therapist | 0.06 (0.07) | 0.32 | .34 |
| Anxiety status | 0.06 (0.18) | 0.07 | .73 |
| 3MFU |  |  |  |
| Sample | -0.24 (0.26) | -0.92 | .37 |
| Therapist | 0.03 (0.06) | 0.15 | .64 |
| Anxiety status | 0.11 (0.16) | 0.14 | .50 |

*Note.* *SE* = standard error; Post = post-CARES assessment; 3MFU = three-month follow-up assessment; Sample = participant classification as Open Trial vs. RCT; Anxiety status = participant classification as Low vs. High anxiety.

**Supplemental Table 10**

*Observed Resolved Means and Standard Deviations for Treatment Outcome Measures Across Assessment Points by Primary and Secondary CU Classification Group*

| Variable | Primary CU (Low Anxiety) | | | | | Secondary CU (High Anxiety) | | | | |
| --- | --- | --- | --- | --- | --- | --- | --- | --- | --- | --- |
|  | Pre | Post-CDI | Post-PDI | Post-CARES | 3MFU | Pre | Post-CDI | Post-PDI | Post-CARES | 3MFU |
|  | *M* (*SD*) | *M* (*SD*) | *M* (*SD*) | *M* (*SD)* | *M* (*SD*) | *M* (*SD*) | *M* (*SD*) | *M* (*SD*) | *M* (*SD)* | *M* (*SD*) |
| ECBI Intensity raw scores | 168.25 (21.10) | 141.48 (24.94) | 117.05 (24.71) | 120.14 (29.13) | 119.52 (29.88) | 190.69  (16.93) | 157.12  (28.23) | 131.94  (24.13) | 134.79  (34.49) | 131.00  (31.31) |
| ECBI Problem raw scores | 22.50 (5.53) | 18.28  (7.44) | 12.61  (7.00) | 11.05  (8.85) | 12.10  (7.97) | 27.71  (4.86) | 21.67  (7.48) | 14.54  (7.34) | 12.17  (8.78) | 12.45  (8.78) |
| CBCL Agg Beh *T* scores | 72.46  (9.28) | 66.68  (10.36) | 61.83  (9.45) | 60.10  (7.38) | 60.84  (9.33) | 83.35  (10.46) | 73.13  (10.30) | 62.23  (6.52) | 64.08  (4.87) | 62.82  (8.02) |
| CBCL Opp Def *T* scores | 70.18  (7.39) | 63.76  (8.48) | 60.26  (8.51) | 59.19  (6.11) | 59.58  (8.66) | 76.82  (4.52) | 68.87  (7.41) | 61.31  (5.85) | 63.17  (6.45) | 64.82  (6.29) |
| CBCL Int *T* scores | 58.82  (9.03) | 59.24  (9.68) | 54.09  (11.46) | 52.19  (10.34) | 52.58  (12.09) | 74.12  (5.59) | 67.60  (6.32) | 63.77  (8.65) | 62.67  (8.29) | 61.55  (6.22) |
| ICU 24-Item Total raw scores | 35.82  (10.90) | 33.71  (9.82) | 30.65  (9.27) | 28.19  (11.30) | 28.21  (11.19) | 39.09  (6.78) | 34.33  (6.43) | 31.00  (5.45) | 28.45  (5.82) | 29.64  (7.38) |
| GEM Affective raw scores | -5.82  (13.04) | -6.74  (12.98) | -3.57  (12.86) | -0.48  (13.86) | -3.25  (13.34) | -3.06  (8.99) | -0.80  (10.23) | 2.69  (11.22) | 0.25  (9.62) | -2.55  (4.70) |

*Note*. Pre = baseline assessment; CDI = Child Directed Interaction; PDI = Parent Directed Interaction; CARES = Coaching and Rewarding Emotional Skills; 3MFU = three month follow-up assessment; *M* = mean; *SD* = standard deviation; ECBI = Eyberg Child Behavior Inventory; CBCL = Child Behavior Checklist; Agg Beh = Aggressive Behavior; Opp Def = Oppositional Defiant Problems; Int = Internalizing; ICU = Inventory of Callous-Unemotional Traits; GEM = Griffith Empathy Measure.

**Supplemental Table 11**

*Observed Mother-Reported Means and Standard Deviations for Treatment Outcome Measures Across Assessment Points by Primary and Secondary CU Classification Group*

| Variable | Primary (Low Anxiety) | | | | | Secondary (High Anxiety) | | | | |
| --- | --- | --- | --- | --- | --- | --- | --- | --- | --- | --- |
|  | Pre | Post-CDI | Post-PDI | Post-CARES | 3MFU | Pre | Post-CDI | Post-PDI | Post-CARES | 3MFU |
|  | *M* (*SD*) | *M* (*SD*) | *M* (*SD*) | *M* (*SD)* | *M* (*SD*) | *M* (*SD*) | *M* (*SD*) | *M* (*SD*) | *M* (*SD)* | *M* (*SD*) |
| ECBI Intensity raw scores | 156.79  (25.56) | 127.92  (28.75) | 103.50  (23.88) | 109.29  (26.58) | 108.71  (28.02) | 178.97  (21.31) | 141.10  (27.27) | 119.48  (25.22) | 121.50  (32.93) | 120.10  (33.47) |
| ECBI Problem raw scores | 20.21  (6.24) | 15.24  (8.24) | 9.74  (6.48) | 9.19  (8.00) | 9.90  (7.64) | 24.35  (6.09) | 16.93  (6.89) | 11.00  (6.65) | 7.83  (6.75) | 9.60  (8.42) |
| CBCL Agg Beh *T* scores | 70.00  (9.20) | 63.76  (10.15) | 58.65  (7.46) | 58.71  (7.04) | 59.63  (8.39) | 79.53  (11.06) | 68.93  (10.27) | 58.46  (7.48) | 61.42  (5.78) | 61.40  (6.96) |
| CBCL Opp Def *T* scores | 69.18  (7.37) | 61.68  (8.90) | 57.48  (7.27) | 58.19  (6.03) | 58.26  (8.07) | 75.18  (5.57) | 66.60  (8.32) | 58.08  (6.56) | 60.58  (6.23) | 61.50  (6.47) |
| CBCL Int *T* scores | 55.75  (8.79) | 55.36  (9.90) | 50.04  (10.20) | 50.52  (10.47) | 50.32  (10.90) | 70.41  (7.32) | 64.13  (7.00) | 59.00  (8.57) | 59.08  (8.48) | 58.70  (7.01) |
| ICU 24-Item Total raw scores | 31.54  (10.23) | 29.39  (10.66) | 24.61  (8.00) | 23.90  (9.83) | 23.16  (10.82) | 35.19  (5.65) | 29.27  (7.81) | 27.00  (5.99) | 24.09  (7.16) | 26.40  (9.41) |
| GEM Affective raw scores | 0.46  (13.55) | 0.34  (14.33) | 5.26  (13.53) | 4.05  (12.14) | 2.70  (13.57) | 2.12  (9.51) | 4.93  (7.27) | 4.46  (10.91) | 1.75  (9.59) | -0.20  (5.33) |

*Note*. Pre = baseline assessment; CDI = Child Directed Interaction; PDI = Parent Directed Interaction; CARES = Coaching and Rewarding Emotional Skills; 3MFU = three month follow-up assessment; *M* = mean; *SD* = standard deviation; ECBI = Eyberg Child Behavior Inventory; CBCL = Child Behavior Checklist; Agg Beh = Aggressive Behavior; Opp Def = Oppositional Defiant Problems; Int = Internalizing; ICU = Inventory of Callous-Unemotional Traits; GEM = Griffith Empathy Measure.

**Supplemental Table 12**

*Observed Father-Reported Means and Standard Deviations for Treatment Outcome Measures Across Assessment Points by Primary and Secondary CU Classification Group*

| Variable | Primary (Low Anxiety) | | | | | Secondary (High Anxiety) | | | | |
| --- | --- | --- | --- | --- | --- | --- | --- | --- | --- | --- |
|  | Pre | Post-CDI | Post-PDI | Post-CARES | 3MFU | Pre | Post-CDI | Post-PDI | Post-CARES | 3MFU |
|  | *M* (*SD*) | *M* (*SD*) | *M* (*SD*) | *M* (*SD)* | *M* (*SD*) | *M* (*SD*) | *M* (*SD*) | *M* (*SD*) | *M* (*SD)* | *M* (*SD*) |
| ECBI Intensity raw scores | 136.78  (25.30) | 115.25  (24.57) | 105.84  (25.57) | 110.36  (30.80) | 104.00  (29.30) | 165.17  (26.05) | 136.50  (33.74) | 115.70  (25.07) | 114.22  (33.65) | 114.67  (28.98) |
| ECBI Problem raw scores | 14.85  (6.32) | 10.10  (6.21) | 7.24  (5.27) | 6.91  (6.41) | 6.42  (5.88) | 21.92  (7.40) | 14.75  (10.74) | 8.45  (7.41) | 9.11  (8.18) | 8.11  (7.32) |
| CBCL Agg Beh *T* scores | 61.50  (9.32) | 57.48  (7.17) | 58.06  (9.94) | 55.27  (6.74) | 57.09  (6.73) | 72.58  (10.82) | 61.45  (8.27) | 56.60  (4.95) | 57.89  (6.31) | 56.00  (6.22) |
| CBCL Opp Def *T* scores | 61.30  (7.87) | 56.67  (7.19) | 56.63  (8.29) | 54.09  (4.35) | 55.64  (6.17) | 70.42  (6.63) | 61.45  (5.68) | 57.40  (5.50) | 57.67  (5.87) | 58.44  (6.27) |
| CBCL Int *T* scores | 51.45  (8.29) | 50.10  (10.76) | 49.19  (10.77) | 44.64  (9.34) | 46.45  (12.82) | 67.00  (4.81) | 59.82  (7.43) | 59.90  (7.13) | 57.11  (7.90) | 54.11  (7.72) |
| ICU 24-Item Total raw scores | 27.84  (9.13) | 25.95  (7.88) | 28.13  (7.44) | 27.62  (8.59) | 26.26  (9.24) | 33.04  (6.93) | 27.81  (8.77) | 23.10  (7.11) | 23.87  (5.05) | 23.89  (6.58) |
| GEM Affective raw scores | 0.05  (10.63) | 0.10  (10.98) | -2.38  (8.47) | -1.73  (9.74) | -1.75  (4.79) | 4.08  (7.60) | 2.75  (13.00) | 11.20  (9.83) | 8.44  (9.55) | 10.00  (10.25) |

*Note*. Pre = baseline assessment; CDI = Child Directed Interaction; PDI = Parent Directed Interaction; CARES = Coaching and Rewarding Emotional Skills; 3MFU = three month follow-up assessment; *M* = mean; *SD* = standard deviation; ECBI = Eyberg Child Behavior Inventory; CBCL = Child Behavior Checklist; Agg Beh = Aggressive Behavior; Opp Def = Oppositional Defiant Problems; Int = Internalizing; ICU = Inventory of Callous-Unemotional Traits; GEM = Griffith Empathy Measure.

**Supplemental Table 13**

*Demographic Characteristics for Completers and Dropouts*

| Variable | Completers | Dropouts |  |
| --- | --- | --- | --- |
|  | *M* (*SD*) | *M* (*SD*) | Significance Test |
| Child age, years | *n* = 34  5.00 (1.10) | *n* = 11  4.35 (0.96) | *t*(43) = -1.78, *p* = .08 |
| Maternal age, years | *n* = 33  37.91 (5.17) | *n* = 11  35.45 (6.46) | *t*(42) = -1.28, *p* = .21 |
| Paternal age, years | *n* = 33  40.52 (6.13) | *n* = 8  39.00 (6.00) | *U* = 160.00, *z* = 0.92, *p* = .37 |
|  | *N* (%) | *N* (%) |  |
| Child sex | *n* = 34 | *n* = 11 | Fisher’s exact test: *p* = .17 |
| Male | 27 (79.4) | 11 (100) |  |
| Female | 7 (20.6) | 0 (0) |  |
| Maternal race/ethnicity | *n* = 34 | *n* = 9 | Fisher’s exact test: *p* = 1.00 |
| White | 26 (76.5) | 7 (77.8) |  |
| Asian, African, self-described other race/ethnicity | 8 (23.5) | 2 (22.2) |  |
| Paternal race/ethnicity | *n* = 34 | *n* = 8 | Fisher’s exact test: *p* = .02* |
| White | 33 (97.1) | 5 (62.5) |  |
| Asian, Pacific Islander | 1 (2.9) | 3 (37.5) |  |
| Parent marital status | *n* = 34 | *n* = 9 | Fisher’s exact test: *p* = .10 |
| In relationship | 31 (91.2) | 6 (66.7) |  |
| Not in relationship | 3 (8.8) | 3 (33.3) |  |
| Annual household income | *n* = 30 | *n* = 8 | Fisher’s exact test: *p* = .71 |
| ≤$150,000 | 16 (53.3) | 5 (62.5) |  |
| >$150,000 | 14 (46.7) | 3 (37.5) |  |
|  | *M* (*SD*) | *M* (*SD*) |  |
|  | *n* = 34 | *n* = 11 |  |
| ECBI |  |  |  |
| Intensity | 176.82 (22.81) | 176.45 (21.81) | *t*(43) = -0.5, *p* = .96, *d* = -0.02 |
| Problem | 24.82 (5.62) | 23.36 (6.56) | *t*(43) = -0.72, *p* = .48, *d* = -0.25 |
| CBCL *T­*-scores |  |  |  |
| Aggressive Behavior | 76.06 (10.80) | 78.18 (12.01) | *t*(43) = 0.55, *p* = .58, *d* = 0.19 |
| Oppositional Defiant | 73.03 (7.17) | 71.64 (7.47) | *U* = 203.50, *z* = 45, *p* = .67, *d* = -0.19 |
| Internalizing | 63.88 (10.65) | 66.82 (11.68) | *t*(43) = 0.78, *p* = .44, *d* = 0.27 |
| ICU Total | 37.09 (9.09) | 36.95 (11.53) | *t*(43) = -0.04, *p* = .97, *d* = -0.01 |
| GEM Affective | -4.55 (11.65) | -5.64 (12.14) | *t*(43) = -0.28, *p* = .78, *d* = -0.10 |

*Note*. ECBI = Eyberg Child Behavior Inventory; CBCL = Child Behavior Checklist; ICU = Inventory of Callous-Unemotional Traits; GEM = Griffith Empathy Measure.

**p* < .05.

**Supplemental Table 14**

*Number (Percentage) of Treatment-Completing Participants in the Normal Range of Functioning at Post-Treatment and Three-Month Follow-Up According to Resolved Parent-Report*

|  | Primary CU (Low Anxiety) | | Secondary CU (High Anxiety) | |
| --- | --- | --- | --- | --- |
| Variable | Post | 3MFU | Post | 3MFU |
|  | *n* = 21* | *n* = 21* | *n* = 12 | *n* = 11^ |
| ECBI Intensity (raw < 131^a^) | 13 (62) | 12 (62) | 6 (50) | 7 (64) |
| ECBI Problem (raw < 15^a^) | 15 (71) | 13 (62) | 8 (67) | 6 (55) |
|  | *n* = 21* | *n* = 19^#^ | *n* = 11^ | *n* = 11^ |
| CBCL Agg (*T* < 70^b^) | 19 (91) | 16 (84) | 11 (92) | 9 (82) |
| CBCL Opp Def (*T* < 70^b^) | 19 (91) | 16 (84) | 9 (75) | 8 (73) |
| CBCL Int (*T* < 64^b^) | 19 (91) | 14 (74) | 4 (33) | 8 (73) |
|  | *n* = 21* | *n* = 19^#^ | *n* = 11^ | *n* = 11^ |
| ICU 24-Item Total (raw < 31^c^) | 12 (57) | 11 (58) | 6 (55) | 4 (36) |

*Note*. Post = post-CARES; 3MFU = three-months follow-up; ECBI = Eyberg Child Behavior Inventory; CBCL = Child Behavior Checklist; Agg = Aggressive Behavior; Opp Def = Oppositional Defiant Problems; Int = Internalizing composite; ICU = Inventory of Callous-Unemotional Traits.

^a^Eyberg & Pincus (1999); ^b^Achenbach & Rescorla (2000; 2001); ^c^Kimonis et al. (2014)

**n* = 1 missing; ^*n* = 1 missing; ^#^*n* = 3 missing

**Supplemental Table 15**

*Results of Model Examining Therapist as Random Factor Predicting Resolved Eyberg Child Behavior Inventory (ECBI) Intensity Raw Scores*

|  | Estimate (*SE*) | 95% CI | *p* |
| --- | --- | --- | --- |
| **Fixed Effects** | | |  |
| Intercept | 141.94 | 135.98 – 147.90 | **<0.001** |
| **Random Effects** | | | |
| σ^2^ | 1186.38 |  |  |
| τ_00,therapist_ | 16.48 |  |  |
| ICC | 0.01 |  |  |
| N_therapist_ | 10 |  |  |
| Observations | 186 |  |  |
| Marginal R^2^ / Conditional R^2^ | 0.000 / 0.014 |  |  |

**Supplemental Table 16**

*Results of Model Examining Therapist as Random Factor Predicting Resolved Eyberg Child Behavior Inventory (ECBI) Problem Raw Scores*

|  | Estimate (*SE*) | 95% CI | *p* |
| --- | --- | --- | --- |
| **Fixed Effects** | | |  |
| Intercept | 16.93 | 15.05 – 18.81 | **<0.001** |
| **Random Effects** | | | |
| σ^2^ | 77.79 |  |  |
| τ_00,therapist_ | 3.27 |  |  |
| ICC | 0.04 |  |  |
| N_therapist_ | 10 |  |  |
| Observations | 186 |  |  |
| Marginal R^2^ / Conditional R^2^ | 0.000 / 0.040 |  |  |

**Supplemental Table 17**

*Results of Model Examining Therapist as Random Factor Predicting Resolved Child Behavior Checklist (CBCL) Aggressive Behavior T-scores*

|  | Estimate (*SE*) | 95% CI | *p* |
| --- | --- | --- | --- |
| **Fixed Effects** | | |  |
| Intercept | 67.56 | 64.93 – 70.20 | **<0.001** |
| **Random Effects** | | | |
| σ^2^ | 118.82 |  |  |
| τ_00,therapist_ | 7.98 |  |  |
| ICC | 0.06 |  |  |
| N_therapist_ | 10 |  |  |
| Observations | 184 |  |  |
| Marginal R^2^ / Conditional R^2^ | 0.000 / 0.063 |  |  |

**Supplemental Table 18**

*Results of Model Examining Therapist as Random Factor Predicting Resolved Child Behavior Checklist (CBCL) DSM-Oriented Oppositional Defiant Problems T-Scores*

|  | Estimate (*SE*) | 95% CI | *p* |
| --- | --- | --- | --- |
| **Fixed Effects** | | |  |
| Intercept | 65.18 | 62.39 – 67.97 | **<0.001** |
| **Random Effects** | | | |
| σ^2^ | 72.15 |  |  |
| τ_00,therapist_ | 12.54 |  |  |
| ICC | 0.15 |  |  |
| N_therapist_ | 10 |  |  |
| Observations | 184 |  |  |
| Marginal R^2^ / Conditional R^2^ | 0.000 / 0.148 |  |  |

**Supplemental Table 19**

*Results of Model Examining Therapist as Random Factor Predicting Resolved Inventory of Callous-Unemotional Traits (ICU) Total Raw Scores*

|  | Estimate (*SE*) | 95% CI | *p* |
| --- | --- | --- | --- |
| **Fixed Effects** | | |  |
| Intercept | 33.18 | 29.48 – 36.88 | **<0.001** |
| **Random Effects** | | | |
| σ^2^ | 74.42 |  |  |
| τ_00,therapist_ | 26.11 |  |  |
| ICC | 0.26 |  |  |
| N_therapist_ | 10 |  |  |
| Observations | 183 |  |  |
| Marginal R^2^ / Conditional R^2^ | 0.000 / 0.260 |  |  |

**Supplemental Table 20**

*Results of Model Examining Therapist as Random Factor Predicting Resolved Griffith Empathy Measure (GEM) Affective Empathy Raw Scores*

|  | Estimate (*SE*) | 95% CI | *p* |
| --- | --- | --- | --- |
| **Fixed Effects** | | |  |
| Intercept | -2.64 | -7.10 – 1.82 | 0.245 |
| **Random Effects** | | | |
| σ^2^ | 112.21 |  |  |
| τ_00,therapist_ | 37.77 |  |  |
| ICC | 0.25 |  |  |
| N_therapist_ | 10 |  |  |
| Observations | 185 |  |  |
| Marginal R^2^ / Conditional R^2^ | 0.000 / 0.252 |  |  |
